# Uptake of oomycete RXLR effectors into host cells by clathrin-mediated endocytosis

**DOI:** 10.1093/plcell/koad069

**Published:** 2023-03-13

**Authors:** Haixia Wang, Shumei Wang, Wei Wang, Lin Xu, Lydia R J Welsh, Marek Gierlinski, Stephen C Whisson, Piers A Hemsley, Petra C Boevink, Paul R J Birch

**Affiliations:** Division of Plant Science, School of Life Sciences, University of Dundee, @James Hutton Institute, Errol Road, Invergowrie, Dundee DD2 5DA, UK; Division of Plant Science, School of Life Sciences, University of Dundee, @James Hutton Institute, Errol Road, Invergowrie, Dundee DD2 5DA, UK; Department of Microbiology and Plant Pathology and Center for Plant Cell Biology, Institute for Integrative Genome Biology, University of California, Riverside, CA 92507, USA; Division of Plant Science, School of Life Sciences, University of Dundee, @James Hutton Institute, Errol Road, Invergowrie, Dundee DD2 5DA, UK; Division of Plant Science, School of Life Sciences, University of Dundee, @James Hutton Institute, Errol Road, Invergowrie, Dundee DD2 5DA, UK; Cell and Molecular Sciences, James Hutton Institute, Errol Road, Invergowrie, Dundee DD2 5DA, UK; Data Analysis Group, Division of Computational Biology, School of Life Sciences, University of Dundee, Dow Street, DD1 5EH Dundee, UK; Cell and Molecular Sciences, James Hutton Institute, Errol Road, Invergowrie, Dundee DD2 5DA, UK; Division of Plant Science, School of Life Sciences, University of Dundee, @James Hutton Institute, Errol Road, Invergowrie, Dundee DD2 5DA, UK; Cell and Molecular Sciences, James Hutton Institute, Errol Road, Invergowrie, Dundee DD2 5DA, UK; Cell and Molecular Sciences, James Hutton Institute, Errol Road, Invergowrie, Dundee DD2 5DA, UK; Division of Plant Science, School of Life Sciences, University of Dundee, @James Hutton Institute, Errol Road, Invergowrie, Dundee DD2 5DA, UK; Cell and Molecular Sciences, James Hutton Institute, Errol Road, Invergowrie, Dundee DD2 5DA, UK

## Abstract

Filamentous (oomycete and fungal) plant pathogens deliver cytoplasmic effector proteins into host cells to facilitate disease. How RXLR effectors from the potato late blight pathogen *Phytophthora infestans* enter host cells is unknown. One possible route involves clathrin-mediated endocytosis (CME). Transient silencing of *NbCHC*, encoding clathrin heavy chain, or the endosome marker gene *NbAra6* encoding a Rab GTPase in the model host *Nicotiana benthamiana*, attenuated *P. infestans* infection and reduced translocation of RXLR effector fusions from transgenic pathogen strains into host cells. By contrast, silencing *PP1c* isoforms, susceptibility factors not required for endocytosis, reduced infection but did not attenuate RXLR effector uptake. Endosome enrichment by ultracentrifugation and sucrose gradient fractionation revealed co-localization of RXLR effector Pi04314-RFP with clathrin-coated vesicles. Immunopurification of clathrin- and NbAra6-associated vesicles during infection showed that RXLR effectors Pi04314-RFP and AvrBlb1-RFP, but not apoplastic effector PiSCR74-RFP, were co-immunoprecipitated during infection with pathogen strains secreting these effectors. Tandem mass spectrometry analyses of proteins co-immunoprecipitated with NbAra6-GFP during infection revealed enrichment of host proteins associated with endocytic vesicles alongside multiple pathogen RXLR effectors, but not apoplastic effectors, including PiSCR74, which do not enter host cells. Our data show that the uptake of *P. infestans* RXLR effectors into plant cells occurs via CME.

## Teaser

Cytoplasmic RXLR effectors from *Phytophthora infestans* enter plant host cells via clathrin-mediated endocytosis.

**IN A NUTSHELL**

**Background:** Disease-causing oomycete pathogens, such as *Phytophthora infestans*, the cause of potato late blight, represent a threat to global food security. To establish disease, *P. infestans* delivers virulence proteins called “RxLR” effectors into living plant cells. RxLR effectors target host proteins and processes and manipulate them to suppress immunity.

**Question:** A key question addressed in our work is: are RxLR effectors taken up into plant host cells by clathrin-mediated endocytosis (CME)?

**Findings:** We explored whether CME is involved in RxLR effector uptake in 3 ways. First, we silenced key host components, clathrin itself and an RAB protein called Ara6, and found that both *P. infestans* infection and RxLR effector delivery into host cells were reduced. Secondly, we used labeled clathrin and Ara6 proteins to immunopurify endosomal vesicles from plant cells during infection and found that a tagged RxLR effector was co-purified. Thirdly, we again captured endosomal vesicles during infection and performed a proteomics study, demonstrating that RxLR effectors, but not “apoplastic” effectors that function outside of plant cells, were associated with the purified endosomes.

**Next steps:** Uptake of RxLR effectors into plant cells by CME implies that, although inside the host cells, the effectors are nevertheless compartmentalized in membrane vesicles. How do they escape the confines of the endomembrane system to reach their host target proteins in different subcellular locations?

## Introduction

Pathogens secrete effector proteins to facilitate infection of their hosts. Plant pathogen effectors can act in the apoplast, outside host cells (Tanaka and Kahmann 2021), or can be delivered to the inside of living plant cells (cytoplasmic effectors) to manipulate host immunity by directly targeting plant proteins (Khan et al. 2018; He et al. 2020). Bacterial pathogens of plants and animals can inject effectors directly into host cells with specialized machinery, such as the type III secretion system (Block and Alfano 2011; Bohn et al. 2019; Hotinger and May 2019; Landry et al. 2020). In addition, bacterial toxins secreted by the type II secretion system can be taken up into host cells via endocytosis (Lauvrak et al. 2004; Torgersen et al. 2005; Sandwig et al. 2010; Johannes 2017). The apicomplexan human parasite *Plasmodium* has been reported to use a translocon to transport effector proteins into the cytoplasm of host erythrocytes (de-Konig-Ward et al. 2009; Ho et al. 2018). More recently, the fungus that causes maize smut disease, *Ustilago maydis*, was shown to form a complex comprising effector and transmembrane proteins that are thought to act as a translocon for cytoplasmic effector delivery (Ludwig et al. 2021). However, the precise means by which filamentous (fungal and oomycete) plant pathogens deliver effectors into plant cells largely remains unknown (Lo Presti and Kahmann 2017; Boevink et al. 2020; Bozkurt and Kamoun 2020).

Oomycetes, such as the potato late blight pathogen *Phytophthora infestans*, cause devastating diseases of crops, threatening food security (Kamoun et al. 2015). During the past 2 decades, considerable progress has been made in understanding the virulence strategies of oomycetes (Wang et al. 2019; Boevink et al. 2020; Bozkurt and Kamoun 2020). Plant pathogenic oomycetes secrete apoplastic effectors, such as protease and glucanase inhibitors, small cysteine-rich (SCR) proteins, including elicitins, and plant cell wall degrading enzymes, all of which are predicted to act outside the plant cell (Wang et al. 2018; Boevink et al. 2020; Bozkurt and Kamoun 2020; Lin et al. 2020). Those oomycetes that engage in a biotrophic phase of interaction with the host, such as *P. infestans*, produce cytoplasmic effectors that are delivered inside plant cells to suppress immunity. Cytoplasmic effectors include the so-called RXLR effectors, with the conserved N-terminal motif Arg-X-Leu-Arg (RXLR, with X being any amino acid) (Rehmany et al. 2005), and the crinkling and necrosis (CRN) effectors, with another conserved N-terminal motif (LXLFLAK) (Haas et al. 2009). The RXLR motif (Whisson et al. 2007; Dou et al. 2008) and LxLFLAK motif (Schornack et al. 2010) have been shown to be responsible for the entry of these effectors into host cells. Following their entry, RXLR and CRN effectors target host proteins that positively or negatively regulate immunity to create a susceptible environment for disease (Wang et al. 2019; He et al. 2020; McLellan et al. 2022). RXLR and apoplastic effectors are secreted from haustoria, finger-like biotrophic structures that form intimate interactions with host cells (Boevink et al. 2020; Bozkurt and Kamoun 2020). Interestingly, whereas apoplastic effectors are secreted by a conventional, brefeldin A (BFA)-sensitive endoplasmic reticulum (ER)-to-Golgi pathway, secretion of RXLR effectors is insensitive to BFA, suggesting a non-conventional route out of the pathogen cell (Wang et al. 2017, 2018). A similar distinction was made between the secretion of apoplastic and cytoplasmic effectors from the rice blast fungus *Magnaporthe oryzae* (Giraldo et al. 2013). The role of the RXLR motif in effector translocation into plant cells is controversial and fiercely debated (Petre and Kamoun 2014). The RXLR motif within *P. infestans* effector Avr3a was demonstrated to be a proteolytic cleavage site, resulting in its removal prior to secretion (Wawra et al. 2017). More recently, a further oomycete cytoplasmic effector, from the fish pathogen *Saprolegnia parasitica*, was reported to be taken into host cells by gp96 receptor-mediated endocytosis, although inhibitor studies suggested that this was clathrin-independent (Trusch et al. 2018).

Endocytosis is a fundamental cellular mechanism that is important for plasma membrane (PM) composition, for regulating the activity of cell surface receptors, and for the uptake of extracellular molecules via a sequence of vesicular compartments (Chen et al. 2011; Fan et al. 2015; Mbengue et al. 2016). Endocytosis thus plays an essential role in plant cell-to-cell communication and in cellular responses to environmental stimuli, including phytohormone and stress signal perception and transduction (Fan et al. 2015; Ekanayake et al. 2019). Similar to animals, there are 2 endocytic pathways in plants: clathrin-mediated endocytosis (CME) and clathrin-independent endocytosis (CIE) (McMahon and Boucrot 2011; Paez-Valencia et al. 2016; Sandvig et al. 2018). CME is the major route for the entry of extracellular molecules into plant cells. CME is initiated by the recruitment of clathrin triskelia composed of clathrin heavy chains (CHCs) and clathrin light chains (CLCs) and the recruitment of adaptor complexes at the PM. Oligomerization of clathrin, together with additional endocytic accessory proteins, generates a lattice-like covering, resulting in clathrin-coated vesicles (CCVs) (Reynolds et al. 2014; Dahhan et al. 2022) that are pinched off from the PM. CCVs further fuse in the cytoplasm with trans-Golgi network/early endosomes (TGN/EE) that are responsible for cargo sorting (Chen et al. 2011; McMahon and Boucrot 2011; Reynolds et al. 2014; Zhang et al. 2015). Clathrin is not immediately lost from endocytic vesicles in plant cells; instead, it is only gradually removed and is still present at the TGN/EE (Narasimhan et al. 2020). Rab GTPases are key regulators of endocytic events at each step (Ebine and Ueda 2009; Minamino and Ueda 2019) and play critical roles in endosomal fusion, regulated by the conformational change between GTP- and GDP-bound forms (Bucci and Chiariello 2006; Schwartz et al. 2007). In addition to the canonical Rab5, which is anchored to the membrane via a geranylgeranyl moiety attached to a C-terminal cysteine motif, plants have evolved the unique Rab GTPase Ara6/RabF1, which is anchored to membranes through dual fatty acylation at its N terminus (Ueda et al. 2001). Arabidopsis (*Arabidopsis thaliana*) ARA6 localizes to the PM, in the TGN, and in multivesicular endosomes (MVEs). It is involved in intracellular endosomal transport (Hoepflinger et al. 2013). The canonical Rab5s (RAB HOMOLOG 1 [RHA1]/RabF2a and Ara7/RabF2b) and plant-specific Rab5 (Ara6/RabF1) localize to distinct populations of endosomes with considerable overlap (Ueda et al. 2004; Haas et al. 2007; Ebine et al. 2011).

CME contributes to plant–microbe interactions and is necessary for endocytosis of pattern recognition receptors (PRRs), such as FLAGELLIN-SENSITIVE 2 (FLS2) and EF-TU RECEPTOR (EFR), that detect the bacterial microbe-associated molecular patterns (MAMPs) flg22 from flagellin and elongation factor Tu (EF-Tu), respectively (Beck et al. 2012; Spallek et al. 2013; Mbengue et al. 2016). In addition, the tomato (*Solanum lycopersicum*) PM-associated Cf4 receptor, which detects the fungal effector Avr4, is also endocytosed by CME following activation (Postma et al. 2016). Interestingly, many endomembrane-trafficking components accumulate at the intimate extrahaustorial membrane (EHM) interface with fungal and oomycete pathogens (Lu et al. 2012; Bozkurt et al. 2014; Dagdas et al. 2016; Inada et al. 2016; Boevink et al. 2020; Bozkurt and Kamoun 2020). Moreover, oomycete effectors contribute to manipulating and rerouting endomembrane-trafficking pathways to the EHM (Bozkurt et al. 2014; Dagdas et al. 2016; Bozkurt and Kamoun 2020). Recently, a report provided evidence that cytoplasmic effectors from *M. oryzae* are taken into host rice (*Oryza sativa*) cells by CME (Oliveira-Garcia et al. 2021), raising the possibility that oomycete RXLR effectors also enter plant cells via this route.

We show here that both clathrin and the plant-specific RAB5 Ara6/RabF1 are evident in vesicle-like structures at the EHM during *P. infestans* interaction with its model host *Nicotiana benthamiana.* Transient silencing of the CHC genes (*NbCHC*) or *NbAra6* significantly attenuated infection and compromised translocation of the RXLR effector Pi04314, tagged with a red fluorescent protein (RFP), into host cells. CLC fused to green fluorescent protein (NbCLC2-GFP) and NbAra6-GFP-associated endosomes were enriched from infection based on ultracentrifugation and immunoprecipitation (IP) assays. We also show that Pi04314-RFP, but not the apoplastic effector PiSCR74-RFP, co-immunoprecipitated with these components. Proteomic analysis of NbAra6-associated endosomes captured during infection revealed (i) an enrichment of plant endosome-related proteins; and (ii) an enrichment of RXLR effectors but not apoplastic effectors from *P. infestans*. Our results, in agreement with the observations for the fungal pathogen *M. oryzae* (Oliveira-Garcia et al. 2021), indicate that cytoplasmic effectors from filamentous plant pathogens exploit CME to enter host cells.

## Results

### Host clathrin and Ara6 are active at haustoria and are required for *P. infestans* infection

**Figure 1.**
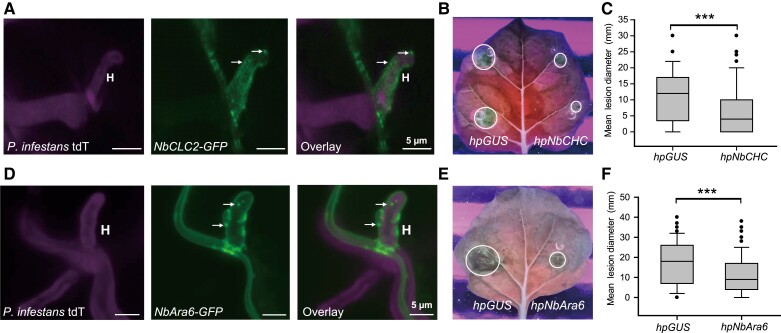
Silencing of *NbCHC* or *NbAra6* reduces *P. infestans* leaf colonization. **A)** NbCLC2-GFP labeled vesicles are observed at haustoria during infection in this representative confocal projection. The image was collected at 5 d post-inoculation (dpi) with sporangia suspension of tdT (tandem-dimer Tomato fluorescent protein)-88069 *P. infestans* transformant in combination with transiently expressed *NbCLC2-GFP*. H, haustoria; white arrows point to example vesicles. **B)** Infection with wild-type *P. infestans* 88069 is compromised in *NbCHC*-silenced leaf areas compared with control areas expressing a *GUS*-silencing construct (*hpGUS*). Example of a leaf image taken at 8 dpi under UV light. **C)** Leaf lesion diameters measured at 8 dpi with sporangia suspension following infiltration of *hpNbCHC2* and control *hpGUS* in each leaf half. One-way ANOVA indicated no significant differences between experimental replicates. Combined data are from 5 experimental replications. A paired 2-tailed *t*-test showed a statistically significant mean difference (***P = 10^–5^, per construct), with a 95% confidence interval of [2.7, 6.6]. **D)** NbAra6-GFP labeled vesicles occur around haustoria during infection. The image was collected at 5 dpi with the tdT-88069 *P. infestans* transformant in combination with transiently expressed *NbAra6-GFP*. H, haustoria. White arrows point to example vesicles. **E)** Infection with wild-type *P. infestans* 88069 is compromised in *NbAra6*-silenced leaf areas compared with control areas expressing *hpGUS*. Example of a leaf image taken at 8 dpi under UV light. **F)** Leaf lesion diameters measured at 8 dpi with sporangia suspension following infiltration of *hpNbAra6* and control *hpGUS* in each leaf half. n=69-way ANOVA indicated no significant differences between experimental replicates. Data were combined from 8 experimental replications. A paired 2-tailed *t*-test showed a statistically significant mean difference (***P = 1.5 × 10^–8^, n=113 per construct), with a 95% confidence interval of [4.2, 8.2].

Clathrin is formed by 3 CHCs and 3 CLCs. The formation of CCVs is caused by CLC-mediated formation of a lattice of CHC trimers referred to as skelia (Kirchhausen 2009; Mettlen et al. 2009). To investigate the potential for involvement of CME in RXLR effector uptake into host cells, we cloned the CLC 2 gene from *N. benthamiana* (*NbCLC2*). We generated GFP fusion constructs to examine clathrin localization during infection. Confocal microscopy indicated the presence of vesicle-like structures labeled by NbCLC2-GFP throughout cells, including around *P. infestans* haustoria during infection (Fig. 1A), suggesting that clathrin could be active at the interface between host and pathogen.

We used a previously described hairpin construct (Mbengue et al. 2016) to silence the 6 genes encoding CHC isoforms in *N. benthamiana* (*NbCHC*) to overcome potential genetic redundancy (Supplemental Fig. S1A). Silencing of *NbCHC* resulted in a significant reduction in FLS2-GFP-associated endosomes triggered by treatment with the bacterial MAMP flg22, compared with a control *hpGUS* silencing construct targeting *β-GLUCURONIDASE* (Supplemental Fig. S1, B and C), as reported previously (Mbengue et al. 2016). In addition, *P. infestans* infection assays showed that colonization of *N. benthamiana* leaves is significantly reduced in *NbCHC-*silenced leaf areas (Fig. 1, B and C) compared with *hpGUS* control areas.

Plant-specific Rab GTPase Ara6/RabF1 is a late endosome (LE)/MVE marker (Minamino and Ueda 2019) and acts as a key regulator of endomembrane-trafficking in plants (Ito and Uemura 2022). In agreement with previous findings (Mbengue et al. 2016), RFP fused to NbAra6 colocalized with flg22-triggered FLS2-GFP-associated endosomes (Supplemental Fig. S2A) in *N. benthamiana*. As observed for NbCLC2-GFP, we detected NbAra6-GFP in vesicle-like structures in cells, including in close proximity to *P. infestans* haustoria (Fig. 1D).

Silencing of *NbAra6* by transient expression of a hairpin construct reduced *NbAra6* expression by >80% (Supplemental Fig. S2B), and significantly reduced the flg22-triggered production of FLS2-GFP-associated endosomes (Supplemental Fig. S2, C and D). As observed for *NbCHC* silencing, *P. infestans* colonization of *N. benthamiana*, measured as lesion diameter, was significantly reduced on plants where *NbAra6* was silenced, compared with the *hpGUS* control (Fig. 1, E and F). Ligand-triggered CME of PRRs, such as FLS2 by flg22 perception, positively regulates sustained PTI responses. As such, knockouts of *CLATHRIN* genes in Arabidopsis resulted in enhanced susceptibility to bacterial pathogens (Mbengue et al. 2016). It is, therefore, surprising that compromised endocytosis, via silencing of either *NbCHC* or *NbAra6*, resulted in significantly reduced susceptibility to *P. infestans,* although it may be hard to directly compare Arabidopsis knockouts with *N. benthamiana-*silenced lines. One possible explanation for our observation, however, is that endocytosis facilitates RXLR effector entry into host cells and that its impairment thus leads to a net reduction in infection. We, therefore, investigated the influence of silencing *Clathrin* and *NbAra6/RabF1* genes on RXLR effector delivery into host cells.

### Silencing *NbCHC* or *NbAra6* attenuates RXLR effector translocation

**Figure 2.**
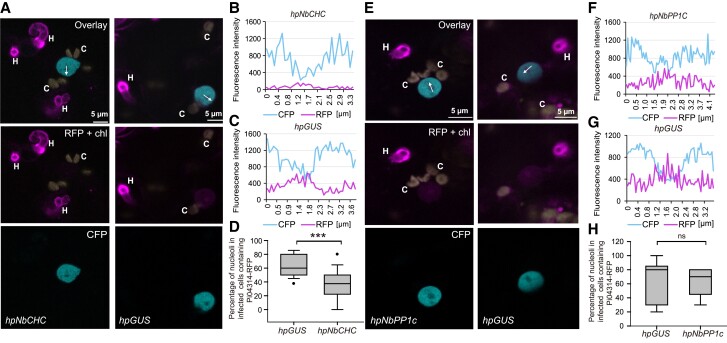
Silencing of *NbCHC* reduces the accumulation of RXLR effector Pi04314 in host nuclei. **A–D)** Silencing of *NbCHC* results in the reduction of Pi04314-mRFP translocation from *P. infestans* haustoria to the host nucleus. **E–H)** Silencing of *NbPP1C* does not affect Pi04314-mRFP translocation from *P. infestans* haustoria to the host nucleus. **A)** and **E)** representative single optical confocal sections of nuclei near haustoria **(H)** in infected cells in which nuclei were labeled by CFP-NbH2B. The nucleolus and nucleoplasm contain red fluorescence, indicating that Pi04314-mRFP has translocated from haustoria to the host cells. Chlorophyll autofluorescence “c” is specifically shown in yellow but also appears in the red channel due to its broad emission spectrum. The white arrows (**A**, *hpCHC* and *hpGUS*) show the lines used for the fluorescence intensity profiles indicated in graphs **(B)** and **(C)**, and the white arrows (**E**, *hpNbPP1c* and *hpGUS*) show the lines used for the fluorescence intensity profiles indicated in graphs **(F)** and **(G)**. **D)** Percentage of host nucleoli containing detectable translocated Pi04314-mRFP among the total observed nuclei in infected cells (with haustoria) in *NbCHC-* and control *GUS*-silenced leaf areas. One-way ANOVA indicated no significant differences between experiments. Combined data were from 7 independent experiments. A paired 2-tailed *t*-test showed a statistically significant mean difference (***P = 2 × 10^–5^, n=15 per construct), with a 95% confidence interval of [18, 38]. **H)** Percentage of host nucleoli containing detectable translocated Pi04314-mRFP among the total observed nuclei in infected cells (with haustoria) in *NbPP1c-* and control *GUS*-silenced leaf areas. ANOVA indicated no significant differences between experiments. Combined data were from 3 independent experiments. A paired 2-tailed *t*-test showed no statistically significant mean difference (ns indicates no significant difference, *P* = 1, n=9 per construct), with a 95% confidence interval of [−17, 17]. The *x*-axis in the graphs represents the distance (in *µ*m) along each white arrow in the corresponding images.

To investigate whether silencing *Clathrin* expression alters RXLR effector translocation into host cells, we silenced *NbCHC* in *N. benthamiana* plants, followed by infection of the silenced plants with transgenic *P. infestans* expressing the RXLR effector fusion construct *Pi04314-RFP*, which was shown to be delivered from haustoria into the host nucleus and nucleolus (Wang et al. 2017). In infected host cells, we detected Pi04314-RFP fluorescence around haustoria, indicating effector secretion. We demonstrated the accumulation of Pi04314-RFP in the nucleolus of haustoriated host cells using transgenic *N. benthamiana* expressing *NbH2B-CFP* (encoding histone H2B fused to cyan fluorescent protein), which fluorescently labels the nucleoplasm (Fig. 2, A–C). The accumulation of Pi04314-RFP in nucleoli was significantly reduced across 103 haustoriated cells silenced for *NbCHC* via *hpNbCHC*, compared with 103 cells expressing the *hpGUS* control (Fig. 2D). We investigated transgenic *P. infestans* expressing another nucleus-targeted RXLR effector as an RFP fusion, Pi22926 (Wang et al. 2018). Again, compared with the *hpGUS* control plants, *NbCHC* silencing resulted in significantly less Pi22926-RFP fluorescence being detected in the host nucleus and nucleolus of cells associated with *P. infestans* haustoria (Supplemental Fig. S3).

For comparison, we investigated the effect of silencing the genes encoding protein phosphatase 1 catalytic subunit (PP1c) isoforms that are targeted by the effector Pi04314, virus-induced gene silencing of which was shown to compromise *P. infestans* infection (Boevink et al. 2016). We confirmed this result by silencing *PP1c* using transient expression of a *hpPP1c* construct (Supplemental Fig. S4). Infection of *N. benthamiana* plants expressing *hpPP1c* was significantly reduced compared with plants expressing the *hpGUS* control (Supplemental Fig. S4, B–D). Silencing of *NbPP1c* using the *hpPP1c* construct did not alter the numbers of FLS2-GFP-associated endosomes following flg22 treatment, compared with the *hpGUS* control, indicating that these PP1c isoforms do not contribute to endocytosis of this PRR (Supplemental Fig. S5). Critically, silencing *NbPP1c* isoforms did not alter the accumulation of Pi04314-RFP in nucleoli of host cells infected by *P. infestans* haustoria (Fig. 2, E–H).

**Figure 3.**
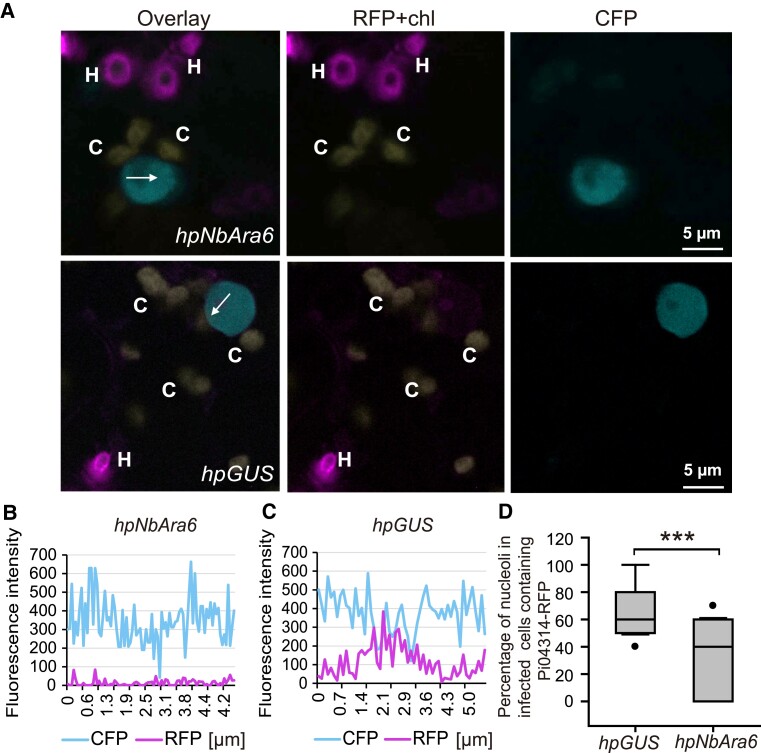
Silencing of *NbAra6* reduces the accumulation of the RXLR effector Pi04314 in host nuclei. **A)** Representative single optical sections of nuclei near haustoria in infected cells in which nuclei were labeled by CFP-NbH2B. The nucleolus and nucleoplasm contain red fluorescence, indicating that Pi04314-mRFP has translocated from haustoria (H) to the host nucleus and nucleolus. Chloroplast autofluorescence “c” is indicated in yellow. The white arrows show the lines used for the fluorescence intensity profiles shown in graphs **(B)** and **(C)**. **D)** The percentage of host nucleoli containing translocated Pi04314-mRFP among the total observed nuclei in infected cells (with haustoria) in control GUS and NbAra6-silenced leaf areas. One-way ANOVA indicated no significant differences between experiments. Combined data were from 3 independent experiments. A paired 2-tailed *t*-test showed a statistically significant mean difference (***P = 8 × 10^–5^, n=18 per construct), with a 95% confidence interval of [20, 48]. The *x*-axis data in the graphs represent the distance (in micrometers) along each white arrow in the images.

We subsequently investigated whether silencing *NbAra6* had any effect upon RXLR effector translocation. Compared with the *hpGUS* control, silencing *NbAra6* with *hpNbAra6* expression resulted in a significant reduction of Pi04314-RFP fluorescence in the host nucleus and nucleolus of cells penetrated by *P. infestans* haustoria (Fig. 3). We observed a significant reduction in the detectable accumulation of Pi04314-RFP in nucleoli (Fig. 3D) across 96 haustoriated cells expressing *hpNbAra6*, compared with 96 cells expressing *hpGUS* control. In agreement, compared with *hpGUS* control plants, *NbAra6* silencing also resulted in significantly less detectable RXLR effector Pi22926-RFP fluorescence in the host nucleus and nucleolus of cells accommodating *P. infestans* haustoria (Supplemental Fig. S6). This observation, coupled with the CHC data, supports the concept of *P. infestans* effector endocytosis being critical to supporting infection. However, as silencing *Clathrin* and *Ara6* is likely to have pleiotropic effects, we investigated the roles of these host proteins in effector uptake by enriching and immunopurifying endocytic vesicles during infection.

### The RXLR effector Pi04314 is co-enriched with clathrin- and Ara6-associated endosomes

**Figure 4.**
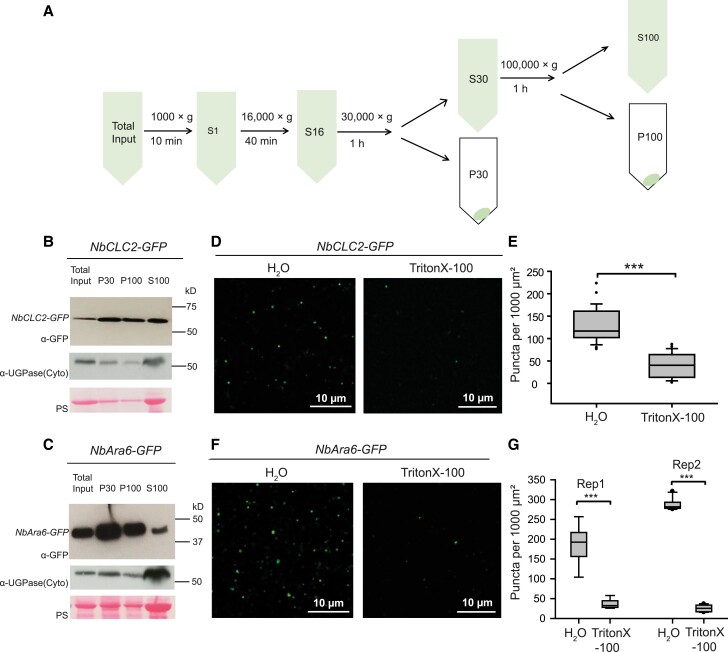
Enrichment of clathrin- or Ara6-associated vesicles analyzed by immunoblot and confocal microscopy. **A)** Scheme of plant endosome isolation by differential ultracentrifugation. **B)** NbCLC2-GFP-positive vesicles and **C)** NbAra6-GFP-positive vesicles analyzed by immunoblot with a GFP antibody (a-GFP). “Total Input” indicates starting material; “P30” or “P100” indicates pellets from 30,000 × *g* or 100,000 × *g* centrifugation. “S100” indicates supernatant from the 100,000 × *g* centrifugation. Cytoplasmic contamination was detected using an antibody for UGPase. “PS” indicates ponceau stain. Numbers indicate size markers (kD). **D)** Puncta of P30 fraction of NbCLC2-GFP; or **F)** of NbAra6-GFP were disrupted by TritonX-100 detergent. Maximum intensity projection images of P30 fractions incubated with H_2_O or 1% TritonX-100 at room temperature for 1 h. **E)** Quantification of NbCLC2-GFP-positive vesicles per 1,000 *μ*m^2^. One-way ANOVA indicated no significant differences between experiments. Combined data were from 2 experimental replicates. Wilcoxon rank sum exact test showed a statistically significant difference of the means (***P = 5 × 10^–13^, n=25 per construct), with a 95% confidence interval of [68, 105]. **F)** Quantification of NbAra6-GFP-positive vesicles per 1,000 *μ*m^2^. One-way ANOVA indicated significant differences between experiments and so both independent experimental replicates are shown. A 2-tailed *t*-test showed a statistically significant difference of the means in both experiments (***P = 4 × 10^–5^, n=6 per treatment for Rep1 and *P* = 2 × 10^–13^, n=10 per treatment for Rep2), with 95% confidence intervals of [130, 230] and [250, 280] for Rep1 and Rep2, respectively.

To further investigate the involvement of CME in Pi04314-RFP uptake into plant cells, we used a series of sequential ultracentrifugation steps to capture clathrin- and NbAra6-associated endosomes from *N. benthamiana* leaf cells (Fig. 4A). Following *NbCLC2-GFP* or *NbAra6-GFP* transient expression in *N. benthamiana*, immunoblots revealed that material pelleted following 30,000 × *g* (P30) or 100,000 × *g* (P100) ultracentrifugation steps is enriched for NbCLC2-GFP (Fig. 4B) or NbAra6-GFP (Fig. 4C), compared with the total leaf protein starting material (Total input). The cytoplasmic protein UDP-glucose pyrophosphorylase (UGPase) (Balsemão-Pires et al. 2011), although still detectable in the P30 and P100 pellets, was highly abundant in the supernatant following 100,000 × *g* (S100), similar to the Ponceau stain for total protein (Fig. 4, B and C). We investigated the material resuspended from P30 (Fig. 4, D–G) or P100 (Supplemental Fig. S7) using confocal microscopy for GFP fluorescence, revealing small vesicle-like structures. The vesicle-like structures were sensitive to treatment with the detergent Triton X-100, suggesting that they are membranous (Fig. 4, D–G; Supplemental Fig. S7). Immunoblotting demonstrated that Triton X-100 treatment of the P30 and P100 fractions does not alter NbCLC2-GFP or NbAra6-GFP protein levels (Supplemental Fig. S7, E and F), indicating that the reduced number of puncta observed was due to disruption by Triton X-100 treatment, rather than loss of protein. To provide independent evidence that these structures are likely to be membranous structures, we assessed whether NbAra6-GFP-associated vesicle-like structures from P30 incorporated the membrane dye FM-4 64 (Supplemental Fig. S8). Importantly, FM-4 64 incubation of the P30 fraction revealed many more vesicular structures than were co-labeled by NbARA6-GFP. This result indicates that NbARA6 labels a subset of isolated membrane compartments, and that compartment identity has been maintained during purification steps.

**Figure 5.**
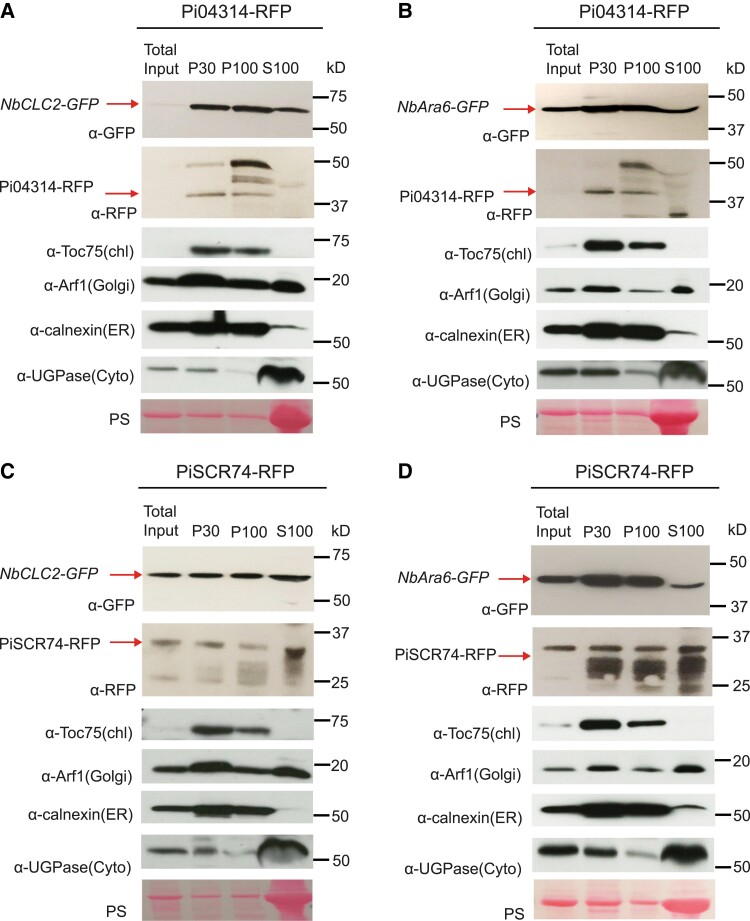
RxLR effector Pi04314 is co-enriched with clathrin- and Ara6-associated vesicles. **A)** and **B)** Plant vesicles isolated by ultracentrifugation from *P. infestans* transformant Pi04314-mRFP-infected leaves transiently expressing NbCLC2-GFP **(A)** or NbAra6-GFP **(B)**. **C)** and **D)** Plant vesicles isolated by ultracentrifugation from *P. infestans* transformant PiSCR74-mRFP-infected leaves transiently expressing NbCLC2-GFP **(C)** or NbAra6-GFP **(D)**. NbCLC2-GFP and NbAra6-GFP were detected by western blot using an antibody against GFP (α-GFP), Pi04314-mRFP and PiSCR74-mRFP were detected by western blot using an antibody against RFP (α-RFP). Chloroplast, Golgi, ER, and Cytoplasm were detected by using antibodies against Toc75 (α-Toc75), Arf1 (α-Arf1), Calnexin (α-Calnexin), and UGPase (α-UGPase), respectively. “PS” indicates ponceau stain. Numbers indicate size markers (kD).

To investigate whether RXLR effectors delivered from *P. infestans* may be co-enriched with clathrin- or Ara6-associated endocytic vesicles, we inoculated transgenic pathogen strains expressing either *Pi04314-RFP* or, as a control, apoplastic effector *PiSCR74-RFP*, onto plants expressing either *NbCLC2-GFP* or *NbAra6-GFP*. Transgenic *Phytophthora* expressing *PiSCR74-RFP* was previously shown to secrete the apoplastic effector at haustoria, but the effector was not detectably taken into plant cells (Lin et al. 2020). Following ultracentrifugation to generate P30 and P100, we clearly detected Pi04314-RFP in these fractions, unlike in the starting Total input material, or in S100, suggesting that it had been co-enriched with NbCLC2-GFP (Fig. 5A) and NbAra6-GFP (Fig. 5B) endocytic vesicles. We detected the PiSCR74-RFP apoplastic effector in all samples (Fig. 5, C and D). Toc75 (TRANSLOCON AT THE OUTER ENVELOPE MEMBRANE OF CHLOROPLASTS 75, a chloroplast outer envelope membrane marker; Eckart et al. 2002), Arf1 (labeling Golgi; Tanigawa et al. 1993; Li et al. 2016), and Calnexin (labeling ER; Howden et al. 2017; Kozlov and Gehring 2020) were also enriched in P30 and P100, compared with S100, whereas cytoplasmic UGPase was again mainly present in S100 samples (Fig. 5, A–D). These results demonstrate that the P30 and P100 fractions from ultracentrifugation do not specifically enrich for endosomes and a number of membranous subcellular compartments are represented, in keeping with the FM-4 64 result (Supplemental Fig. S8).

**Figure 6.**
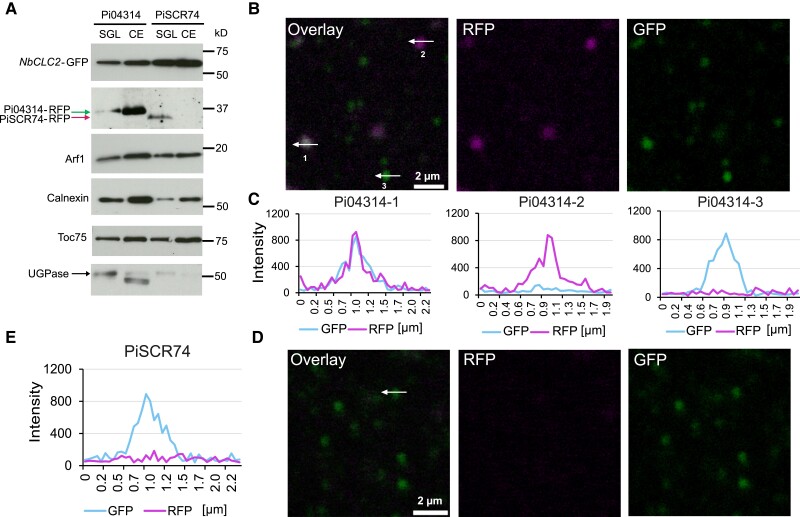
Co-localization of *P. infestans* effector Pi04314-mRFP and NbCLC2-GFP-positive vesicles. **A)** NbCLC2-GFP labeled vesicles and Pi04314-mRFP are detectable in SGL and CE fraction, but PiSCR74-mRFP was not detectable in CE by immunoblot. NbCLC2-GFP was detected by immunoblotting using an antibody against GFP (α-GFP), and Pi04314-mRFP and PiSCR74-mRFP were detected using an antibody against RFP (α-RFP). Chloroplast, Golgi, ER, and cytoplasmic markers were detected with antibodies against Toc75 (α-Toc75), Arf1 (α-Arf1), Calnexin (α-Calnexin), and UGPase (α-UGPase), respectively. Numbers indicate size markers (kD). **B)** Representative single optical sections of NbCLC2-GFP- and Pi04314-mRFP-labeled vesicles. The numbered white arrows show the lines used for the fluorescence intensity profiles shown in graph **(C)**. **D)** Representative single optical sections of NbCLC2-GFP- and PiSCR74-mRFP-labeled vesicles. The white arrow shows the line used for the fluorescence intensity profile shown in graph **(E)**. The *x*-axis in the graphs represents the distances (in *µ*m) along each white arrow in the images.

To verify that the RXLR effector Pi04314-RFP detected in immunoblots in Fig. 5 is associated with vesicles, we performed a bottom-loaded discontinuous sucrose gradient (8.5% to 25% to 35% to 42%, w/w) (Urbanska et al. 2011). The immunoblots indicated that following infection of plants expressing *NbCLC2-GFP* with transgenic *P. infestans* secreting either *Pi04314-RFP* or *PiSCR74-RFP*, we detected both effectors in the sucrose gradient loading (SGL) material (62% [w/-w] sucrose was added to the resuspended P30 to make 1 mL of 40.6% [w/w] sucrose samples called SGL). We detected Pi04314-RFP in the interphase of 25% to 35% sucrose that was reported to include endosomes (crude endosome [CE] fraction) (Urbanska et al. 2011; de Araújo et al. 2015). We also detected the control endomembrane compartment markers calnexin, Arf1, and Toc75, whereas we barely detected PiSCR74-RFP and the cytoplasmic marker UGPase in the CE sample (Fig. 6A). We inspected the CE fraction by confocal microscopy; of 544 vesicles labeled with NbCLC2-GFP tested, following infection with *P. infestans* secreting Pi04314-RFP, 26 also showed Pi04314-RFP fluorescence (4.8%) (Fig. 6, B and C). By contrast, of 657 vesicles labeled with NbCLC2-GFP tested following infection with *P. infestans* secreting PiSCR74-RFP, none were positive for PiSCR74-RFP-associated fluorescence (Fig. 6, D and E). These results demonstrate that Pi04314-RFP, but not PiSCR74-RFP, can co-localize with host CCVs during infection with *P. infestans* lines secreting these effector fusions.

**Figure 7.**
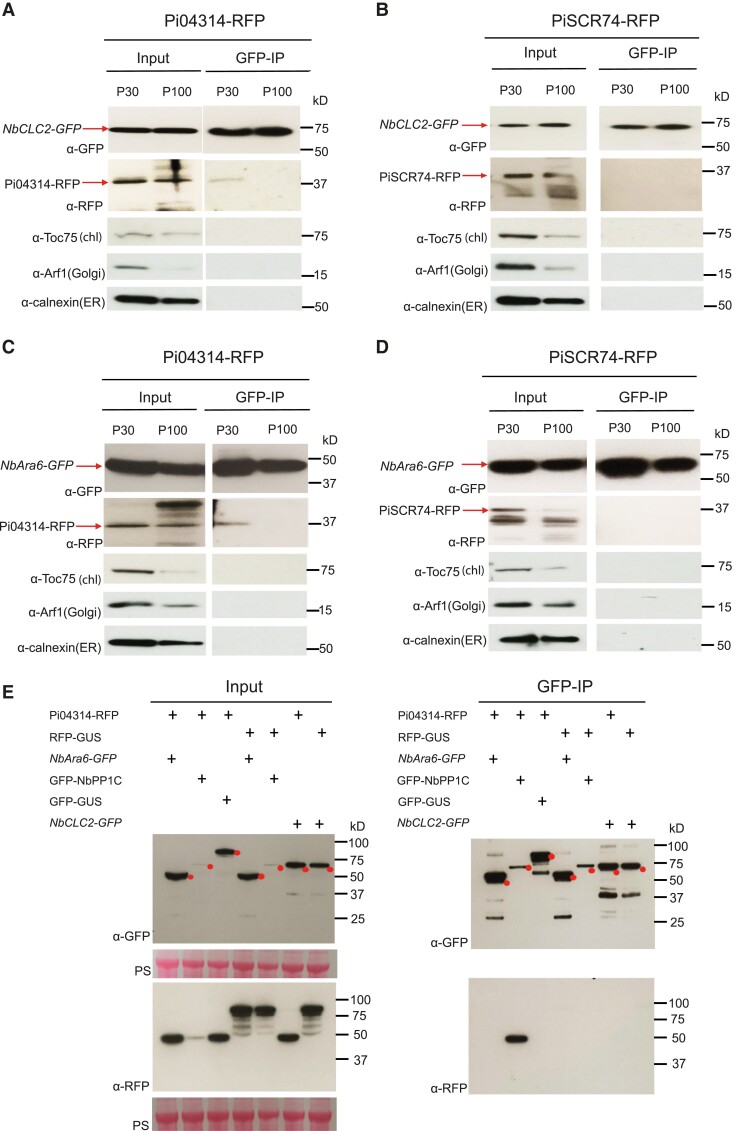
The RxLR effector Pi04314 is co-immunoprecipitated with clathrin- and Ara6-associated vesicles. **A)** and **B)** Plant vesicles isolated by ultracentrifugation from leaves expressing *NbCLC2-GFP* and infected with *P. infestans* transformants expressing *Pi04314-mRFP***(A)** or *PiSCR74-mRFP***(B)**. **C)** and **D)** Plant vesicles isolated by ultracentrifugation from leaves expressing *NbAra6-GFP* and infected with *P. infestans* transformants *Pi04314-mRFP***(C)** or *PiSCR74-mRFP***(D)**. IP samples were purified NbCLC2-GFP- and NbAra6-GFP-positive vesicles in the P30 and P100 samples after incubation with GFP-Trap beads. NbCLC2-GFP and NbAra6-GFP were detected by immunoblot using an antibody against GFP (α-GFP); Pi04314-mRFP and PiSCR74-mRFP were detected by immunoblot using an antibody against RFP (α-RFP). Chloroplast membrane, Golgi, and ER membrane were detected with antibodies against Toc75 (α-Toc75), Arf1 (α-Arf1), and Calnexin (α-Calnexin), respectively. **E)** Protein–protein interactions in planta between NbCLC2-GFP and Pi04314-mRFP, NbAra6-GFP and Pi04314-mRFP were detected by immunoblot. GFP-NbPP1C was used as a positive control, and GFP-GUS and RFP-GUS were used as negative controls. IP samples were plant lysates incubated with GFP-Trap beads. NbCLC2-GFP, NbAra6-GFP, GFP-NbPP1C, and GFP-GUS were detected with an antibody against GFP (α-GFP), and Pi04314-mRFP and RFP-GUS were detected with an antibody against RFP (α-RFP). “PS” indicates ponceau stain. Numbers indicate size markers (kD).

To purify NbAra6 or NbCLC2-associated vesicles and eliminate contamination from other endomembrane compartments, we performed IP assays. The ultracentrifugation steps from leaves expressing *NbCLC2-GFP* (Fig. 7, A and B; Supplemental Fig. S9) or *NbAra6-GFP* (Fig. 7, C and D; Supplemental Fig. S9), infected with *P. infestans* transgenic lines expressing either *Pi04314-RFP* or *PiSCR74-RFP*, again revealed the presence of both effectors (Fig. 7, A–D; Supplemental Fig. S9). In addition, we also detected calnexin, Arf1, and Toc75 in material pelleted by ultracentrifugation (Fig. 7, A–D), as shown previously (Fig. 5). Following IP with an anti-GFP antibody to capture NbCLC2-GFP (Fig. 7, A and B; Supplemental Fig. S9) or NbAra6-GFP (Fig. 7, C and D; Supplemental Fig. S9), we failed to detect these endomembrane compartment markers (Fig. 7) or the apoplastic effector fusion PiSCR74-RFP (Fig. 7; Supplemental Fig. S9). By contrast, Pi04314-RFP co-immunoprecipitated with both NbCLC2-GFP (Fig. 7A; Supplemental Fig. S9A) and NbAra6-GFP (Fig. 7C; Supplemental Fig. S9C), especially from resuspended P30 material.

As Pi04314 is an RXLR effector that targets proteins within host cells (Boevink et al. 2016), it was formally possible that its presence within NbCLC2-GFP or NbAra6-GFP immunoprecipitates was due to a direct interaction between the effector and these host proteins as virulence targets after being delivered into the plant cell. We thus co-expressed a version of *Pi04314-RFP*, without its signal peptide, in *N. benthamiana* cells together with *NbCLC2-GFP*, *NbAra6-GFP*, or a construct encoding its known target *NbPP1c-1-GFP* (Boevink et al. 2016) and performed IP with an anti-GFP antibody. Whereas Pi04314-RFP co-immunoprecipitated with NbPP1c-1-GFP, as anticipated, it was not co-immunoprecipitated with either NbCLC2-GFP or NbAra6-GFP (Fig. 7E). This result demonstrates that there is no direct interaction between Pi04314 and either of these endosome-associated proteins in the plant cytoplasm, and thus confirms that the capture of Pi04314-RFP with NbCLC2-GFP (Fig. 7A) and NbAra6-GFP (Fig. 7C), especially from P30, is likely due to its presence within endosomes associated with these plant fusion proteins.

### Endosome proteome enrichment following NbAra6-GFP IP

**Figure 8.**
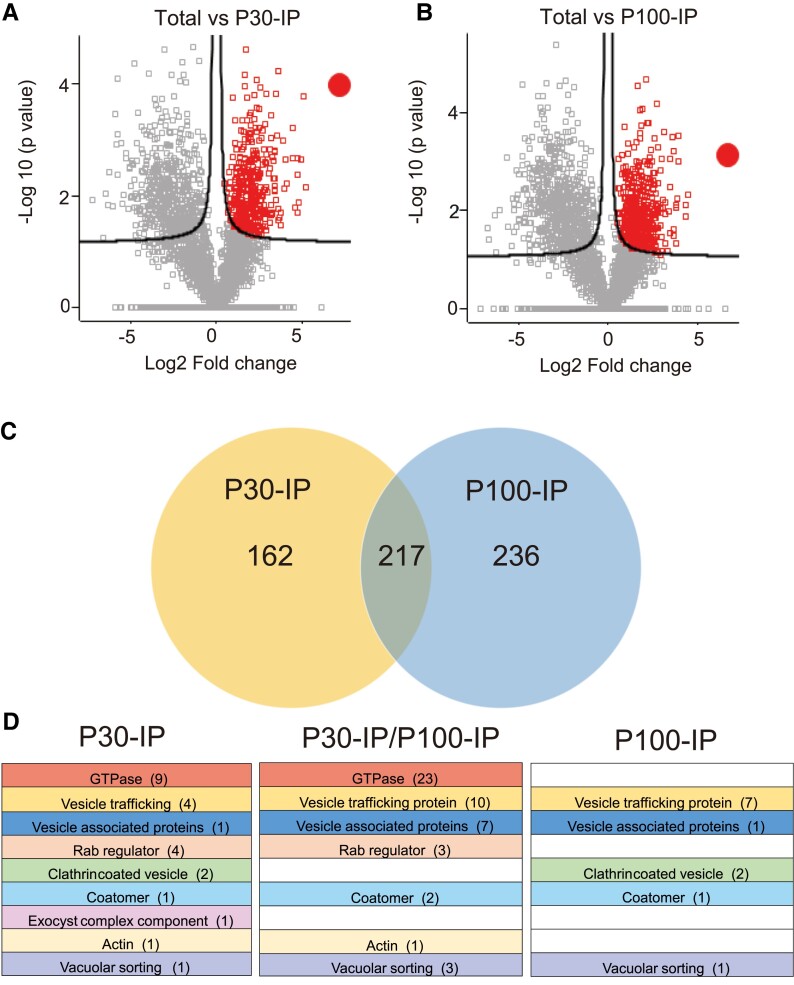
Plant vesicle proteome enrichment following NbAra6-GFP IP. **A)** Volcano plots comparing P30-IP or **B)** P100-IP with Total input sample proteomes. The *x*-axis represents the fold change in label-free quantitation intensity of proteins between samples, and the *y*-axis shows the statistical significance of the differences. Red squares indicate enriched sequences. The red-filled circles indicate enriched NbAra6-GFP. **C)** Venn diagram comparison of significantly enriched proteins (*t*-test, *P* < 0.05) in P30-IP and P100-IP proteomes. Numbers in each area of the Venn diagram indicate the numbers of protein sequences. **D)** Colors and names represent different protein categories associated with endosomes (detailed in Supplemental Data Set 2, C–E). Columns labeled P30-IP or P100-IP contain proteins only enriched in P30-IP or P100-IP samples. The column labeled P30-IP/P100-IP represents enriched proteins in common between P30-IP and P100-IP. Numbers in brackets indicate the numbers of independent proteins represented in each endosome category (Supplemental Data Set 2, C–E).

Encouraged by the successful enrichment of NbAra6-GFP-positive endosomes that incorporated the membrane dye FM4-64 (Supplemental Fig. S8) and were sensitive to Triton X-100 (Fig. 4), coupled with the co-IP of NbARA6-GFP with the RXLR effector fusion Pi04314-RFP from transgenic *P. infestans* infections of *N. benthamiana* (Fig. 7), we performed an independent study of the endosome proteome during infection. We focused on *N. benthamiana* leaves transiently expressing *NbAra6-GFP* that were densely spot-inoculated with water droplets containing zoospores and sporangia from wild-type *P. infestans* line 88069. Following endosome enrichment (Fig. 4A) and NbAra6-GFP IP (Fig. 7) from P30 and P100 fractions in triplicate, we analyzed samples by tandem mass spectrometry (MS/MS). All *N. benthamiana* proteins identified following these IPs, as well as those identified following MS/MS of the corresponding “Total input” samples, are listed in Supplemental Data Set 1. Volcano plots revealed 379 *N. benthamiana* proteins that were significantly enriched (false discovery rate [FDR] = 0.05) in the NbAra6-GFP P30-IP compared with the starting Total input material (Fig. 8A; Supplemental Data Set 2A), and 453 proteins were enriched in the P100 IP compared with Total input (Fig. 8B; Supplemental Data Set 2B), in both cases including NbAra6 itself. Gene ontology (GO) term enrichment analyses revealed that among the proteins enriched in the P30-IP and P100-IP samples were those involved in small GTPase-mediated signal transduction and a variety of transport steps (Supplemental Fig. S10).

A comparison revealed that, of the total proteins enriched in the independent P30 and P100 NbAra6-GFP IPs, 217 were common (Fig. 8C). Endosome-associated proteins previously observed from affinity purification of fluorescently tagged Rab proteins in Arabidopsis (Heard et al. 2015) were also enriched here using NbAra6-GFP. Some of the enriched endosome proteins were shared between the P30 and P100 IP samples, and some were exclusive to one or the other (Fig. 8D; Supplemental Data Set 2, C–E). This result confirms that P30 and P100 ultracentrifugation followed by NbAra6-GFP IP significantly enriches endosome-associated proteomes.

### RXLR effector proteins are detected in the NbAra6-associated proteome

**Figure 9.**
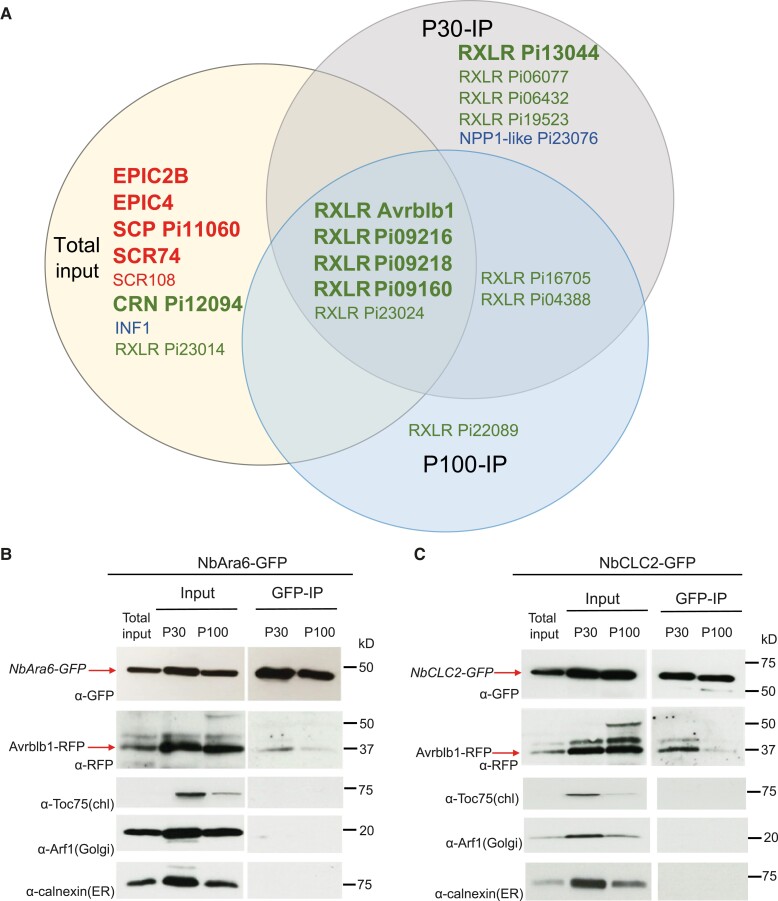
Enrichment of RxLR effectors in P30 and P100 NbAra6-GFP IP samples verified for Avrblb1. **A)** Venn diagram showing *P. infestans* cytoplasmic effectors and apoplastic proteins detected in P30-IP, P100-IP, and Total input samples. Proteins indicated are detected in at least 2 replicates in a given category (Total input, P30-IP, or P100-IP). Red text indicates secreted apoplastic effectors from *P. infestans*. Blue text indicates *P. infestans* secreted proteins detected as MAMPs. Green text indicates secreted RxLR and CRN candidate cytoplasmic effectors. Large bold font indicates the most abundant sequences (>2 peptides in >2 replicates; Supplemental Data Set 4C). Smaller font indicates effectors detected by single peptides in at least 2 replicates from a category. Plant vesicles isolated by ultracentrifugation from leaves expressing NbAra6-GFP **(B)** or NbCLC2-GFP **(C)** infected with *P. infestans* transformants expressing *Avrblb1-mRFP*. IP samples were NbAra6-GFP- or NbCLC2-GFP-positive vesicles purified from the P30 and P100 samples after incubation with GFP-Trap beads. NbCLC2-GFP and NbAra6-GFP were detected by immunoblot using an antibody against GFP (α-GFP) and Pi04314-mRFP by α-RFP. Chloroplast membrane, Golgi, and ER membrane were detected with antibodies against Toc75 (α-Toc75), Arf1 (α-Arf1), and Calnexin (α-Calnexin), respectively. Numbers indicate size markers (kD).

To aid in the identification of *P. infestans* proteins in the Total input and NbAra6-GFP immunoprecipitated endosome proteomes during infection, we generated parallel independent MS/MS data in triplicates from Total input and from P30 and P100 enrichments and NbAra6-GFP IP of uninfected leaves. Peptide matches to *P. infestans* proteins identified from infected samples were retained if they were above the identity threshold using Mascot (Supplemental Data Set 3). We focused on secreted *P. infestans* proteins with known or likely functions in host interactions: cytoplasmic and apoplastic effectors and MAMPs. Peptides matching these proteins were absent in the uninfected samples following predicted proteome screens for *N. benthamiana* and *P. infestans* sequences. Due to the relatively low biomass of pathogen compared with plants during the biotrophic phase of infection, and the small size of most effector proteins, we only registered single peptide matches to a given protein (Supplemental Data Set 4, A and B). Overall, we identified peptide matches to 44 RXLR effector candidates, 7 CRN effector candidates, 8 apoplastic effector candidates, and 6 secreted proteins that are detected by the host as MAMPs, from at least one experimental replicate of Total input, P30 NbAra6-GFP IP, or P100 NbAra6-GFP IP (Supplemental Data Set 4C). Those present in at least 2 of the 3 replicates of a given category (Total, P30, or P100) are shown in Fig. 9A. Based on these criteria, we observed that, whereas we mainly detected apoplastic effectors (EPICs, SCRs, and SCP), including PiSCR74, and the abundant elicitin INF1, in Total input samples, we detected most RXLR effectors in Total, P30 NbAra6-GFP IP, and P100 NbAra6-GFP IP, or exclusively in the P30 NbAra6-GFP IP and P100 NbAra6-GFP IP samples, or in P30 or P100 NbAra6-GFP IPs alone (Fig. 9A). One RXLR effector candidate (Pi23014/CRE16) and 1 candidate CRN effector (Pi12094) were found exclusively in 2 or more Total input replicates, whereas the NPP1-like protein (Pi23076) was present in multiple replicates of P30 NbAra6-GFP IP (Fig. 9A). Overall, we detected 12 RXLR effectors in 2 or more P30 NbAra6-GFP IP and/or P100 NbAra6-GFP IP replicates, 7 of which were not detected in Total input samples (Fig. 9A).

To provide independent verification of proteins emerging from the proteomics analyses, we generated a *P. infestans* transformant expressing the RXLR effector fusion *PiAvrblb1-RFP*, demonstrating that the effector is secreted from haustoria and is translocated into plant cells to accumulate in the nucleus and nucleolus (Supplemental Fig. S11) during infection, as anticipated (Wang et al. 2019). IP with an anti-GFP antibody revealed that PiAvrblb1-RFP co-immunoprecipitates with NbAra6-GFP (Fig. 9B) and NbCLC2-GFP (Fig. 9C), strongly from P30-IP samples and weakly from P100 IP samples, consistent with MS/MS results. This result supports the observations that CME is a route of host cell entry for RXLR effectors from the oomycete *P. infestans*.

## Discussion

We provide independent lines of evidence showing that *P. infestans* cytoplasmic RXLR effectors enter host plant cells via CME. Silencing of *NbCHC*, encoding CHC, or the LE/MVE marker *NbAra6/RabF1*, each led to reduced *P. infestans* infection (Fig. 1) and a concomitant reduction in translocation of RXLR effector fusion proteins Pi04314-RFP and Pi22926-RFP from transgenic pathogen strains into the host nucleus, their known site of activity (Figs. 2 and 3; Supplemental Figs. S3 and S6). Enrichment of clathrin- and NbAra6-associated endosomes from infected leaves, via ultracentrifugation (Figs. 4 and 5) and IP (Fig. 7), led to co-IP of the RXLR effector Pi04314, but not the apoplastic effector PiSCR74. Sucrose gradients allowed us to demonstrate that Pi04314, but not PiSCR74, was associated with clathrin-labeled vesicles (Fig. 6). Finally, analyses of the proteomes of NbAra6-positive endosomes from leaves infected with non-transgenic *P. infestans* strain 88069 revealed the enrichment of endosome-associated plant proteins (Fig. 8) and the detection of RXLR effectors in endosome fractions, whereas apoplastic effectors and the abundant elicitin INF1 were detected in total leaf protein but were largely absent from enriched endosome material (Fig. 9). Each of these observations is discussed below.

### Clathrin and Ara6 contribute to *P. infestans* infection and effector translocation

Both *N. benthamiana* CLC-2 (NbCLC2) and NbAra6/RabF1 labeled vesicle-like structures throughout cells, including in close proximity to the *P. infestans* haustorial interface (Fig. 1). This result is consistent with previous studies that have reported localization of host endocytic components at the EHM (Bozkurt and Kamoun 2020), including Rab7 and the Rab5 family member Ara7 during oomycete infections (Lu et al. 2012). An additional study showed Ara6/RabF1 also localizes to the haustorial interface between Arabidopsis and the biotrophic powdery mildew fungal pathogen *Golovinomyces orontii* (Inada et al. 2016). More recently, the rice clathrin component OsCLC-1 was shown to localize to the biotrophic interfacial complex (BIC) formed at the site of cytoplasmic effector delivery from the fungal pathogen *M. oryzae* (Oliveira-Garcia et al. 2021).

A study by Mbengue et al. (2016) demonstrated that, whereas a knockout of *chc2* in Arabidopsis did not prevent acute FLS2-mediated immune responses, such as the production of reactive oxygen species, or early flg22-triggered activation of mitogen-activated kinases (MAPKs), it did lead to reduced callose deposition and to enhanced susceptibility to the bacterial pathogen *Pseudomonas syringae* (Mbengue et al. 2016). By contrast, *Clathrin* gene silencing here, and also silencing of *NbAra6,* the product of which is associated with endosomes (Supplemental Fig. S2), led to reduced *P. infestans* colonization of *N. benthamiana* leaves (Fig. 1). In agreement, Arabidopsis *chc2-1* and *chc2-2* knockout mutants were more resistant to the biotrophic fungus *Golovinomyces cichoracearum* and showed enhanced activation of MAPKs and callose accumulation (Wu et al. 2015). Moreover, silencing of *CLATHRIN* in rice, or the chemical inhibition of its encoded protein with cantharidin, significantly attenuated infection by *M. oryzae* (Oliveira-Garcia et al. 2021). Thus, whereas knockout of *Clathrin* increased susceptibility to bacterial infection (Mbengue et al. 2016), it compromised infection by oomycete (Fig. 1) and fungal (Wu et al. 2015; Oliveira-Garcia et al. 2021) pathogens, suggesting that the net effect of clathrin association with *P. infestans* haustoria (Fig. 1), the *M. oryzae* BIC (Oliveira-Garcia et al. 2021), or *G. cichoracearum* penetration sites (Wu et al. 2015), is beneficial to the respective pathogens. One possible explanation is that silencing *Clathrin*, or the endosome marker *NbAra6*, while potentially reducing sustained PTI signaling, also compromised delivery of cytoplasmic effectors from the pathogens into host cells to suppress immunity.

Transgenic *P. infestans* strains expressing *Pi04314-RFP* or *Pi22926-RFP* secrete these RXLR effectors at haustoria and deliver them to host nuclei and nucleoli (Wang et al. 2017, 2018), their known sites of activity (Boevink et al. 2016; Ren et al. 2019). Silencing of *NbCHC* or *NbAra6,* but not a *hpGUS* control, significantly reduced Pi04314-RFP (Figs. 2 and 3) and Pi22926-RFP (Supplemental Figs. S3 and S6) accumulation in host nucleoli. Importantly, whereas silencing *NbCHC* and *NbAra6* compromised flg22-triggered FLS2 endocytosis (Supplemental Fig. S1; Fig. 2), silencing *NbPP1c* isoforms did not. This result is consistent with clathrin and NbAra6, but not NbPP1c isoforms, contributing to Pi04314-RFP uptake into host cells via CME. These observations are in agreement with CME providing a route for the delivery of RXLR effectors into host plant cells. However, silencing of endocytic components can have pleiotropic effects upon infection (Ekanayake et al. 2019). CCVs are involved in altering the levels and activities of PM components during plant infection (Ekanayake et al. 2019) and they have been implicated in positively regulating (e.g. Mbengue et al. 2016) or in negatively regulating (e.g. Wu et al. 2015; Yao et al. 2022) plant immunity. It was thus important to capture endosomes during infection to investigate the presence of cytoplasmic effectors, consistent with their delivery into host cells via CME.

### *P. infestans* RXLR Pi04314 is associated with host endosomes

We adapted previously described methods (Reynolds et al. 2014; Heard et al. 2015) to enrich plant endosomes (Fig. 4A). Following 30,000 × *g* (P30) and 100,000 × *g* (P100) ultracentrifugation of material extracted from *N. benthamiana* expressing *NbCLC2-GFP* or *NbAra6-GFP*, we observed green fluorescent vesicle-like structures using confocal microscopy. These structures were sensitive to the detergent Triton X-100, which is consistent with these structures including NbCLC2-GFP- and NbAra6-GFP-positive endosomes (Fig. 4; Supplemental Fig. S7). The membrane dye FM4-64 co-stained these NbAra6-GFP-associated structures, indicating they were likely to be vesicular (Supplemental Fig. S8). FM4-64 stained more vesicle-like structures than those labeled with NbAra6-GFP (Supplemental Fig. S8), indicating that a range of vesicles was enriched. These vesicles may include secretory vesicles from the pathogen in addition to various plant vesicles, including some formed as a result of cell damage. Indeed, antibodies to a range of endomembrane compartment markers, including calnexin (ER), Arf1 (Golgi apparatus), and Toc75 (chloroplast outer membrane), indicated that these markers were enriched by P30 and P100 ultracentrifugations (Fig. 5). Moreover, they were also evident in a CE fraction following ultracentrifugation through a sucrose gradient (Fig. 6). When homogenizing plant tissue, all membranes, including the Golgi, ER, and chloroplasts, break into small fragments that can then reseal themselves to form small vesicles (e.g. Castle 2003). We thus anticipate that some such damage-associated vesicles are of a size to co-migrate to a similar sucrose density as endosomes. These limitations for purifying endosomes using sucrose gradients led Drakakaki et al. (2012) to propose the use of immuno-isolation as a superior method.

Despite the limitations of sucrose gradients for endosome purification, they nevertheless do capture vesicular material and the CE fraction revealed co-localization of Pi04314-RFP with NbCLC2-GFP fluorescence in vesicle-like structures. Recently, a detailed study of CCV formation, kinetics, and dynamics showed that, unlike in yeast (*Saccharomyces cerevisiae*) and animals, the clathrin component CLC2-GFP fusion protein is often retained from the earliest stage of endocytosis until the EE/TGN (Narasimhan et al. 2020). It was thus not surprising to see a range of CLC2-GFP-coated vesicle sizes in Fig. 6, all of which were within the size ranges reported by Narisimhan et al. (2020). Of 544 NbCLC2-GFP labeled vesicles tested from the CE fraction following infection with *P. infestans* secreting Pi04314-RFP, 26 also revealed Pi04314-RFP fluorescence (4.8%) (Fig. 6, B and C), whereas none of the 657 CE-associated NbCLC2-GFP labeled vesicles showed RFP fluorescence following infection with *P. infestans* secreting PiSCR74-RFP (Fig. 6, D and E). The absence of NbCLC2-GFP/PiSCR74-RFP co-localization agrees with the effector not being detected in corresponding CE immunoblots (Fig. 6A) and is consistent with it not entering host cells. The low level (4.8%) of Pi04314-RFP co-localization with NbCLC2-GFP is also not surprising, given that most CCVs are unlikely to be from infected cells or, indeed, to be in close proximity to haustoria. Interestingly, some Pi04314-RFP-labeled vesicles from the CE fraction were not also labeled with NbCLC2-GFP (Fig. 6C). These vesicles could be other classes of vesicles in the host endocytic pathway, after clathrin uncoating, or could be secretory vesicles derived from the pathogen, as discussed above.

The most specific procedure for purifying diverse subtypes of vesicles is immunopurification as established in animal cells (Steuble et al. 2010; Jeppesen et al. 2019) and recently successfully applied in plants (He et al. 2021; Suzuki et al. 2021, 2022) in combination with differential ultracentrifugation. We, therefore, performed IP with an anti-GFP antibody on samples enriched from plants expressing *NbCLC2-GFP* or *NbAra6-GFP* and confirmed that each of these was immunoprecipitated, whereas abundant intracellular membrane-associated markers such as calnexin, Arf1, and Toc75 were largely excluded (Figs. 7 and 9). In the case of plants infected with *P. infestans* expressing apoplastic effector PiSCR74-RFP, the effector was not co-immunoprecipitated following IP for NbCLC2-GFP or NbAra6-GFP, although it was observed in P30 and P100 vesicle enrichments. By contrast, the RXLR effector fusions Pi04314-RFP and AvrBlb1-RFP were co-immunoprecipitated from plants infected with *P. infestans* expressing these effectors following IP for NbCLC2-GFP or NbAra6-GFP (Figs. 7 and 9). Interestingly, Pi04314-RFP and AvrBlb1-RFP were primarily detected in the NbCLC2-GFP and NbAra6-GFP IPs from P30. Given that Pi04314-RFP (Fig. 5) and AvrBlb1-RFP (Fig. 9) were clearly co-enriched in P30 and P100 with NbCLC2-GFP or NbAra6-GFP, the detection of these effectors, and indeed of PiSCR74-RFP, in P100 enrichment may be due to the presence of other classes of vesicles in that material, including ones derived from the pathogen, as indicated above. However, the clear enrichment of Pi04314-RFP and AvrBlb1-RFP in P30 and P100 (Figs. 5 and 9), followed by their co-IP with either NbCLC2-GFP or NbAra6-GFP (Figs. 7 and 9), especially from P30, supports the hypothesis that they are taken into host cells via CME.

RXLR effectors such as Avr3a have been reported to bind to phosphoinositides via positively charged amino acids in the effector domain (Yaeno et al. 2011). If Avr3a represents a general rule, this finding raises the question: might RXLR effectors bind to phosphoinositides on the membranes of endosomes during protein extraction? However, a separate report (Wawra et al. 2012) indicated that the Avr3a effector domain only binds phosphoinositides when the protein is not properly folded, i.e. it is an artifact associated with denatured protein. In Fig. 7E, we demonstrated that RXLR effector Pi04314, when present within plant cells, can be co-immunoprecipitated with its target, PP1c, but not following IP of either NbAra6-GFP or NbCLC2-GFP, proportions of which will be associated with phosphoinositide-rich outer surfaces of endomembrane compartments. The co-IP in Fig. 7E does not support the idea that Pi04314 associates with phosphoinositide-rich endosome membranes during protein extractions. Moreover, only 4.8% of CCVs from the CE preparations shown in Fig. 6 were associated with Pi04314-mRFP, again suggesting that the RXLR effector does not associate with phosphoinositides during protein extractions.

### *P. infestans* RXLR effectors are enriched in the host endosome proteome

Following NbAra6-GFP IP from P30 and P100, MS/MS revealed a total of 615 proteins that were statistically enriched (Supplemental Data Set 2) compared with the Total input samples, including 217 in common between P30-IP and P100-IP (Fig. 8). Remarkably, 86 of these proteins, 50 of which are shared between P30 and P100, are annotated as plant endosome-associated proteins, demonstrating that the IP of NbAra6-GFP likely did capture entire endosomes. Interestingly, some endosome-associated proteins were specifically enriched in P30 or P100 samples, indicating the potential for both common and distinct NbAra6-GFP-positive vesicles to have been enriched by the different ultracentrifugation steps (Fig. 8; Supplemental Data Set 2). This is perhaps consistent with the differential co-IP of Pi04314-RFP from P30 and P100 samples (Fig. 7), suggesting that vesicles with which this effector is associated are more prevalent in P30. It is worth noting that the initial P30 enrichment contains more large vesicles, potentially including LEs or multivesicular bodies, than the subsequent P100 enrichment (Fig. 4; Supplemental Fig. S7).

The low pathogen biomass and small size of secreted proteins from *P. infestans* that interact with plant cells meant that few peptides were detected for these proteins. Nevertheless, we detected peptides from 12 RXLR effectors in 2 or more replicates from P30 NbAra6-GFP IP or P100 NbAra6-GFP IP. Six of these were also represented in Total input, whereas 6 were found only in P30 and P100, or P30 alone (Fig. 8). Two or more peptides per sample for 5 RXLR effectors, including Avrblb1/ipiO1 whose encoding gene is known to be highly expressed during infection (Pieterse et al. 1994), were detected in 2 or more P30 replicates (Fig. 8). These observations are consistent with these effectors entering host cells via CME. Moreover, they are consistent with the observed co-IP of Pi04314-RFP mainly from NbCLC2-GFP and NbAra6-GFP IPs from P30 enrichments (Fig. 6; Supplemental Fig. S9).

In striking contrast to the RXLR effectors, we detected 5 apoplastic effectors and the abundant elicitin INF1 in 2 or more replicates only of Total input, often as multiple independent peptides (Fig. 8; Supplemental Table S2C). This result is in agreement with these proteins not entering plant cells. Indeed, PiSCR74-RFP was independently shown not to co-IP with NbCLC2-GFP or NbAra6-GFP from P30 and P100 (Fig. 6: Supplemental Fig. S9), consistent with reports that it is active in the apoplast (Lin et al. 2020) rather than within plant cells. Interestingly, we detected 1 CRN effector and 1 RXLR effector, albeit only as a single peptide for the latter, exclusively in 2 or more replicates of Total input. It is possible that either these candidate effectors do not enter plant cells, or that they do so by means other than CME, or via NbAra6-independent endosomes. Remarkably, we detected peptides exclusively in 2 P30 replicates from a member of the NPP1-like protein (NLP) family, which is conserved across kingdoms of life and may contribute to cytolytic activity by forming pores in host membranes (Ottman et al. 2009) (Fig. 8; Supplemental Data Set 2). Further work is needed to confirm whether this protein does indeed enter plant cells via CME and to determine whether it is cytolytic.

We provide independent lines of evidence to demonstrate that oomycete RXLR effectors enter host plant cells via CME. This view is consistent with the report by Oliveira-Garcia et al. (2021) showing that cytoplasmic effectors from the fungal pathogen *M. oryzae* also enter host cells via CME. Whereas we previously reported that the RXLR effector Pi04314 from *P. infestans* only enters plant cells in the presence of the pathogen (Wang et al. 2017), another oomycete cytoplasmic effector, SpHtp3 from the fish pathogen *S. parasitica*, was reported to enter host cells in a pathogen-independent fashion (Trusch et al. 2018). SpHtp3 entered host cells via lipid-raft-dependent CIE, using the inhibitor nystatin, and entry was associated with the conserved receptor gp96 (Trusch et al. 2018). There is thus potentially more than one route of entry for oomycete cytoplasmic effectors into host cells.

A key question for future research to address is: how are the RXLR effectors released from endosomes to reach their targets at a range of subcellular destinations in the host cell? While retrograde trafficking through the TGN and the ER is a known route for bacterial toxins (Sandivig et al. 2010), potential vesicle fusion events, or vesicle disruption, may also be possible. Knowledge of the importance of CME to the entry of cytoplasmic effectors from filamentous pathogens into plant cells may inform the development of new approaches to prevent disease by these economically important pathogens.

## Materials and methods

### Plant materials

Wild-type *N. benthamiana* and a transgenic line in which the nucleus is labeled with histone H2B tagged with a cyan fluorescent protein (CFP-NbH2B; Goodin et al. 2007) were grown in a glasshouse at 22 °C on general purpose compost under long-day conditions of 16-h light/8-h dark, supplemental sodium light intensity of 130 to 150 *μ*E m^−2^ s^−1^, and 40% humidity to 4-5 wk old.

### Cloning and construct generation

All primer sequences are listed in Supplemental Table S1. To create the hairpinNbAra6 construct targeting *NbAra6a* (Niben101Scf29276g00003.1 from Sol Genomics Network) and *NbAra6b* (Niben101Scf00271g01020.1 from Sol Genomics Network, https://solgenomics.net/), a region of *NbAra6b* (379 to 594 bp) was amplified by PCR using cDNA as a template. To generate the negative control *hairpinGUS* (*hpGUS*) construct, a fragment of the bacterial *GUS* gene (CP093368.1 from NCBI, 142 to 715 bp) was amplified by PCR using a GUS-GFP plasmid as a template. The PCR fragments were recombined into entry vector pDONR201 (Invitrogen). To generate *hpNbCHC*, *hpNbAra6*, *hpNbPP1c*, and *hpGUS*, pTOPO-D.NbCHC (Mbengue et al. 2016), pDONR201.NbhpAra6, pDONR201.NbPP1C (Boevink et al. 2016), and pDONR201.hpGUS were recombined with pHellsgate8 (Helliwell et al. 2002), respectively. *NbAra6-GFP* and *NbCLC2-GFP* were generated by recombining pDONR201.NbAra6b (Niben101Scf00271g01020.1) and pDONR201.NbCLC2 (Niben101Scf01101g05008.1), which were generated by recombining the respective sequences amplified by PCR into the entry vector, into pB7FWG2. *FLS2-GFP* (Hurst et al. 2018) was created by recombining pENTR D-TOPO containing the *FLS2* coding sequence without a stop codon with pK7FWG2 (Karimi et al. 2002), and *AtAra6-RFP* was created by recombining pENTR. AtAra6 (At3g54840.1; Latijnhouwers et al. 2005) was cloned into a modified pMDC83 (Curtis and Grossniklaus 2003) in which *GFP* was replaced with *mRFP*. All constructs were generated by Gateway cloning, following the manufacturer's instructions (Invitrogen). The coding sequence without the stop codon of *Avrblb1* (PITG_21388; GenBank NCBI accession EEY61733) was amplified from the genomic DNA of *P. infestans* WT 3928A using gene-specific primers and cloned into the pPL-RAG vector for *P. infestans* transformation as described previously (Wang et al. 2017).

### *Agrobacterium tumefaciens* transient assays

All constructs were transformed into Agrobacterium (*A. tumefaciens*) strain GV3101 pMP90 (Koncz et al. 1989), Agrobacterium cells were grown in yeast extract and beef medium containing the appropriate antibiotics at 28 °C overnight. Overnight cultures were centrifuged at 3,214 × *g* for 10 min at room temperature, and pellets were resuspended in infiltration medium (10 mM 2-(*N*-morpholino) ethanesulfonic acid, 10 mM MgCl_2_, and 150 mM acetosyringone) and incubated in the dark for at least 1 h before infiltration into leaves using a 1-mL syringe after needle wounding. Bacterial suspensions were adjusted to an optical density at 600 nm (OD600) of 0.25 for transient silencing assays. For transient co-expression assays, *N. benthamiana* leaves were pressure-infiltrated with a mixture of Agrobacteria harboring the *NbAra6*-*GFP* construct at a final OD600 of 0.005 or *NbCLC2-GFP* at a final OD600 of 0.01, *AtFLS2-GFP* at a final OD600 of 0.5, *AtAra6-RFP* at a final OD600 of 0.1, and the silencing suppressor p19 (Voinnet et al. 1999; Scholthof 2006) at a final OD600 of 0.01.

### Quantitative RT–PCR

Total RNA was extracted from the leaves of *N. benthamiana*-silenced plants using an RNeasy plant mini kit (74904, Qiagen) according to the manufacturer's instructions. First-strand cDNA was synthesized using a SuperScript II Reverse Transcriptase kit (18064014, Invitrogen) and quantitative PCR (qPCR) was carried out using SYBR green (A25918, Thermo Fisher Scientific) as described previously (McLellan et al. 2013). Primers for qPCR are listed in Supplemental Table S1 and gene expression levels were analyzed using the comparative Ct method as described by Livak and Schmittgen (2001) and Cikos et al. (2007).

### *P. infestans* inoculation

*P. infestans* wild-type strain 88069 and transgenic strains expressing *Pi04314-mRFP*, *Pi22926-mRFP*, *PiSCR74-mRFP*, and *Avrblb1-mRFP*, in addition to cytoplasmic *GFP* (Wang et al. 2017, 2018; Lin et al. 2020) (for simplicity these strains are referred to as Pi04314-RFP, PiSCR74-RFP, and Avrblb1-RFP), or tandem-dimer Tomato fluorescent protein (tdT) were grown for 2 wk at 19 °C on rye agar plates with 100 *µ*g mL^−1^ ampicillin, 10 *µ*g mL^−1^ geneticin, and 0.001% (w/v) pimaricin before sporangia were harvested by flooding with sterile distilled water (SDW), scraping with a plastic spreader, and filtering through a 70-*μ*m nylon cell-strainer (Corning) to remove hyphae. The resulting suspension was centrifuged at 1,598 × *g* for 10 min at room temperature. Sporangia from the wild-type strain 88069 were resuspended in SDW to 100,000 sporangia per mL for *P. infestans* virulence assays. Sporangia from the transgenic strains Pi04314-RFP, Pi22926-RFP, and Avrblb1-RFP were resuspended in SDW to 500,000 sporangia per mL for effector translocation observations, and 1 to 2 *P. infestans* inoculum drops (10 *μ*L per drop) were inoculated on either half of an *N. benthamiana* leaf at 2 d post Agrobacterium infiltration; 2 to 3 leaves were inoculated from 5 to 7 plants per experimental replicate. For plant endosome isolation, a large number of 10-*μ*L aliquots of *P. infestans* inoculum was drop inoculated over the leaf surface with 5 mm space at a final density of 120,000 sporangia per mL of 88069, or 500,000 sporangia per mL of transformants Pi04314-RFP, PiSCR74-RFP, and Avrblb1-RFP, and infected leaves were collected at 3 to 4 d post-inoculation.

### Flg22 peptide and Triton X-100 treatments

Either half of *FLS2-GFP* transiently expressing *N. benthamiana* leaf was infiltrated with 100 *μ*M flg22 or control H_2_O for 80 min before imaging, as described previously (Mbengue et al. 2016); 1 to 2 leaves with treatments and 5 to 15 different cell areas were observed under a confocal microscope per experimental replicate. For the detergent disruption of endosomes, 5 *μ*L 1% (v/v) Triton X-100 (Sigma) was added to 45 *μ*L suspensions of the P30 or P100 fractions and incubated for 1 h at room temperature before imaging; 2 to 3 samples with treatments and 4 to 5 different areas were observed per experimental replicate.

### Confocal microscopy analyses

*N. benthamiana* leaf pieces expressing fluorescently tagged proteins were mounted on slides and imaged using a Nikon A1R confocal microscope with a 40× water immersion lens. CFP was imaged with 457 nm excitation and emissions were collected between 465 and 500 nm. RFP was imaged using 561 nm excitation and emissions were collected between 570 and 620 nm. Chlorophyll autofluorescence was imaged with 514 nm excitation and emissions collected between 663 and 738 nm. GFP was imaged with 488 nm excitation and emissions collected between 500 and 530 nm. For imaging translocation of effectors, saturation of the RFP signal at haustoria was unavoidable due to the comparatively low fluorescence from the translocated fusion protein in the plant cell nuclei. Plant endosome-associated fluorescence in ultracentrifuged fractions was imaged with a 60× water immersion lens. The pinhole was set to 1.2 Airy units for the longest wavelength fluorophore of any combination. For fluorescence quantification of internalized FLS2-GFP puncta in plant cells or of NbAra6-GFP- and NbCLC2-GFP-positive bodies in endosome fractions, the green puncta were counted using the ImageJ (https://imagej.net/software/fiji/downloads) software plugin Analyze-Cell counter. Image processing for figures was conducted with Adobe ILLUSTRATOR CS5.1. To study co-localization of NbCLC2-GFP and Pi04314-mRFP, the pinhole was set to 0.8 Airy units for the longest wavelength fluorophore of the combination. The green puncta were counted using NIS-Elements AR 4.30 Manual measurement Counts. Intensity profiles were used to determine the co-localization of green and red puncta to exclude the coincidental overlap of green and red vesicles.

### Plant endosome isolation

Plant endosomes were isolated from 20 g *N. benthamiana* leaves (Fig. 4A) with 40 mL isolation buffer (150 mM Na-HEPES pH 7.5, 10 mM EDTA, 10 mM EGTA, 17.5% [w/w] sucrose, 7.5 mM KCl,10 mM DTT, 1× PIC-W, 1×PIC-D, and 1×E-64) (Reynolds et al. 2014; Heard et al. 2015). The cellular debris in the total input was removed by filtration through Nylon Filter Mesh 100 (Sigma) and a 50-*μ*L aliquot was removed as a “Total input” sample, the remaining lysate was centrifuged at 1,000 × *g* and 4 °C for 10 min (Centrifuge: Eppendorf 5810/5810R). Supernatants were transferred into pre-chilled ultracentrifugation tubes and further purified by centrifugation for 40 min at 16,000 × *g* using a fixed-angle 50.2 Ti Rotor and polycarbonate thick wall centrifuge tubes (25 × 89 mm) with an Optima L-80XP Ultracentrifuge (all from Beckman Coulter) at 4 °C. The supernatants were transferred to new ultracentrifuge tubes and centrifuged for 1 h at 30,000 × *g* to obtain the P30 pellets. The supernatants of P30 were centrifuged for 1 h at 100,000 × *g* to obtain the P100 pellets. Pellets were fully resuspended in 500 *μ*L isolation buffer by constant rotation for 1 h at 4 °C. Forty-five microliters of resuspension of each pellet was boiled with 45 *μ*L 2× SDS-loading buffer at 95 °C for 10 min for preparation of the P30 and P100 samples for immunoblotting. Proteins in the 1.6-mL supernatants of P100 were precipitated with 4 times the sample volume of pre-chilled acetone overnight at −20 °C then centrifuged for 10 min at 16,000 × *g* and 4 °C. These pellets were boiled with 45-*μ*L 2× SDS-loading buffer at 95 °C for 10 min for preparation of the S100 samples for immunoblotting.

### Fractionation of endosomes by ultracentrifugation using sucrose gradients

Optimized endosome fractionation on a step sucrose gradient was based on Urbanska et al. (2011). In brief, the well-resuspended P30 sample was adjusted to 40.6% (w/w) sucrose by mixing with 62% (w/w) sucrose. One milliliter of this mix was loaded at the bottom of a 13.2-mL ultracentrifuge tube. It was overlaid with 3.3 mL 35% (w/w) sucrose, 3.3 mL 25% (w/w) sucrose, and 3.3 mL 8.5% (w/w) sucrose to fill the tube. The loaded gradient was centrifuged in an SW 41 Ti rotor (Beckmann Coulter) at 210,000 × *g* for 3 h at 4 °C. The 25% to 35% interphase was collected and was pelleted at 100, 000 × *g* for 1 h at 4 °C for microscopy analysis and immunoblotting. All sucrose solutions contained protease inhibitors 1× PIC-W, 1× PIC-D, and 1× E-64.

### Immunopurification of endosomes

For NbAra6-GFP and NbCLC2-GFP labeled endosome immuno-isolation, 20 *μ*L GFP magnetic protein A beads (Chromotek) or Binding control magnetic beads (Chromotek) were washed with isolation buffer 3 times. To pre-clear the suspensions, 20 *μ*L of the Binding control beads were added into the 1-mL suspensions of P30 or P100, followed by 1 h constant rotation at 4 °C. These cleared suspensions of P30 or P100 were transferred into new tubes and 20 *μ*L of the GFP beads were added, followed by overnight incubation at 4 °C with constant rotation. Bead-bound endosome material was collected and washed with isolation buffer 5 times for 2 min at 4 °C with constant rotation (without 1× PIC-W, 1× PIC-D, and 1× E-64). The bead-bound endosomes were boiled with 45 *μ*L 2× SDS-loading buffer and proteins were resolved in an SDS-PAGE gel.

### Immunoblotting

For the analysis of protein interaction in planta, protein fusions transiently overexpressed at 2 dpi in *N. benthamiana* and proteins were extracted using protein extraction buffer: GTEN buffer (10% [v/v] glycerol; 25 mM Tris-HCl, pH 7.5; 1 mM EDTA; 150 mM NaCl) containing 1 mM phenylmethylsulfonyl fluoride (PMSF), 10 mM DTT, 0.5% (v/v) Nonidet P40, and protease inhibitor tablet (04693132001, Roche). Protein lysates were incubated on ice for 30 min followed by 10 min 13,800 × *g* centrifugation at 4 °C. These cleared supernatants were transferred into new tubes and 20 *μ*L of GFP beads were added, followed by 1 h incubation at 4 °C with constant rotation. Bead-bound proteins were collected and washed with washing buffer 5 times (GTEN buffer with 1 mM PMSF) and were boiled with 45 *μ*L 2× SDS-loading buffer at 95 °C for 10 min. Samples were loaded onto 12% SDS-PAGE gels and run for 2 h at 120 V. Proteins were transferred onto nitrocellulose membrane for 2 h at 30 V with Ponceau staining to demonstrate transfer and loading. Membranes were blocked in 4% (w/v) nonfat dry milk in 1× phosphate-buffered saline Tween 20 (PBST, 137 mM NaCl; 12 mM phosphate; 2.7 mM KCl, pH 7.4; 0.2% [v/v] Tween 20) for 1 h before the addition of primary antibodies overnight. Primary antibodies: anti-GFP (11814460001, Sigma) raised in mouse at 1:3,300 dilution; anti-RFP (5f8-100, Chromotek) raised in rat at 1:2,000 dilution; and anti-Toc75 (AS08 351, Agrisera), anti-Arf1 (AS08 325, Agrisera), anti-calnexin (AS12 2365, Agrisera), and anti-UGPase (AS05086, Agrisera) raised in rabbit at 1:5,000 dilution. Horseradish peroxidase-conjugated secondary antibodies: anti-mouse (A9044, Sigma), anti-rabbit (11814460001, A8275), and anti-rat (ab6836, Abcam) at 1:5,000 dilution.

### Liquid chromatography-coupled tandem mass spectrometry identification of proteins of NbAra6-GFP-positive endosomes

Three independent biological replicates were performed to generate “Total input” and NbAra6-GFP-positive endosome immunopurification samples. Samples were resolved via 1 D SDS-PAGE and gels were stained with Quick Coomassie Stain. Each gel lane was cut into 4 fragments of 1 to 2 cm each (corresponding to approximately 8 to 15, 15 to 25, 25 to 40, and 40 to 260 kD) then subjected to in-gel digestion with 1 mg mL^−1^ Trypsin (Thermo) at a final concentration of 12.5 *µ*g mL^−1^. Digested peptides were run on a Q-Exactive Plus (Thermo Scientific) instrument coupled to a Dionex Ultimate 3000 HPLC system (Thermo Scientific). A 2% to 35% (v/v) gradient comprised of eluent A (0.1% [v/v] formic acid) and eluent B (80% [v/v] acetonitrile/0.1% [v/v] formic acid) was used to run a 120-min gradient with each fragment run consecutively and blanks used between samples. The top 10 most intense peaks from a mass range of 350 to 1,600 *m*/*z* in each MS1 scan with a resolution of 70,000 were then taken for MS2 analysis at a resolution of 17,500. Spectra were fragmented using higher-energy C-trap dissociation.

### Proteomic data analysis

MS/MS data were analyzed using MaxQuant version 1.6.6.0 against the *N. benthamiana* database (Kourelis et al. 2019). The MS/MS–flow-injection mass spectrometry tolerance was set to 20 ppm, and the ion trap mass spectrometer match tolerance was 0.15. Fixed modifications included carbamidomethyl (C), and variable modifications included oxidation (M); a maximum value of 2 missed cleavages was used. The protein FDR was set to 0.01. The minimum peptide length was 7. Output data from MaxQuant were analyzed using Perseus_2.0.3.0, LFQ (Label-free quantification). LFQ intensity data were transformed to log_2_(LFQ) and were filtered based on the following criteria (min valids = 2, Mode = in at least 1 group). A 2-way *t*-test was used for analyzing differentially abundant proteins in the 2 samples being compared. The significantly enriched proteins in P30-IP and P100-IP samples were categorized on the basis of GO annotation using NiBen v101 protein ID (Sol Genomics Network) and analysis tools AgriGO v2.0 (Tian et al. 2017) and Revigo (Supek et al. 2011). *P. infestans* peptides were searched using Mascot Daemon against the *P. infestans* uniprot-proteome_UP000006643 (https://www.uniprot.org/proteomes/UP000006643). Peptide mass tolerance was set to 10 ppm, fragment mass tolerance was set to 0.06 kD, and maximum missed cleavages were set to 2. RXLR proteins annotated to lack signal peptide and/or RXLR motif and the CRN proteins lacking signal peptide and/or LFLAK motif were excluded from Fig. 9 and Supplemental Data Set 3C.

### Statistical analysis

Data from independent experimental replications were combined when one-way ANOVA indicated no significant differences between them; otherwise, they were analyzed separately. Statistical significance between 2 conditions was determined using a paired *t*-test, as appropriate for paired measurements coming from the same leaf. Both tests were performed using R-4.2.0 (https://cran.r-project.org). Boxplots were created with SigmaPlot 14.5 (https://systatsoftware.com/sigmaplot). The lower and upper sides of the box in the boxplots within figures are the lower quartile (Q1) and upper quartile (Q3), and the boxes cover the interquartile ranges (IQRs). The horizontal line that splits the box into 2 is the median. The whiskers are the 2 lines outside the box, which go from the lower quartile to the minimum (Q1-1.5×IQR) and from the upper quartile to the maximum (Q3 + 1.5×IQR).

### Accession numbers

Gene accession numbers are given throughout the methods section. NbAra6a: Niben101Scf29276g00003.1; NbAra6b: Niben101Scf00271g01020.1; NbCLC2: Niben101Scf01101g05008.1; AtAra6: At3g54840.1; Avrblb1: PITG_21388; GUS: CP093368.1.

Proteomics data have been submitted to the PRIDE repository: https://www.ebi.ac.uk/pride/markdownpage/citationpage, Project accession number: PXD040285. Raw confocal microscope images containing metadata are deposited on the Dundee University “Discovery” repository (https://doi.org/10.15132/10000193). In addition, unprocessed MaxQuant and Mascot proteomics data are included on the Discovery repository at https://doi.org/10.15132/100001934.

## Supplementary Material

koad069_Supplementary_Data

## Supplemental data

**Supplemental Figure S1**. Silencing of *NbCHC* reduces flg22-induced endocytosis of FLS2-GFP.

**Supplemental Figure S2**. Silencing of *NbAra6* reduces flg22-induced endocytosis of FLS2-GFP.

**Supplemental Figure S3**. Silencing of *NbCHC* reduces RXLR effector Pi22926 accumulation in host nuclei.

**Supplemental Figure S4**. Leaf colonization by *P. infestans* is significantly reduced in susceptibility factor *NbPP1C*-silenced plants.

**Supplemental Figure S5**. Silencing of *NbPP1C* does not alter flg22-triggered endocytosis of FLS2-GFP.

**Supplemental Figure S6**. Silencing of *NbAra6* reduces RXLR effector Pi22926 accumulation in host nuclei.

**Supplemental Figure S7**. P100 enriched puncta are disrupted by Triton X-100 detergent.

**Supplemental Figure S8**. Puncta of NbAra6-GFP enriched in P30 fraction stained with FM4-64.

**Supplemental Figure S9**. RxLR effector Pi04314 is co-immunoprecipitated with clathrin- and Ara6-associated vesicles.

**Supplemental Figure S10**. GO enrichment analysis of proteins significantly enriched in P30-IP and P100-IP following NbAra6-GFP immunopurification.

**Supplemental Figure S11**. Secretion and translocation of RxLR effector AVRblb1 during infection.

**Supplemental Table S1**. Primers used in this study.

**Supplemental Data Set 1**. *N. benthamiana* proteins detected in Total input, P30-IP, and P100-IP proteomes.

**Supplemental Data Set 2**. Plant proteins significantly enriched in P30-IP and P100-IP proteomes.

**Supplemental Data Set 3**. *P. infestans* proteins detected in the plant-interaction Total input, P30-IP, and P100-IP proteomes.

**Supplemental Data Set 4**. *P. infestans* virulence determinants detected in the plant-interaction Total input, P30-IP, and P100-IP proteomes.

**Supplemental Data Set 5**. Summary of statistical analyses.
