# Supplementary material for: Uptake of oomycete RXLR effectors into host cells by clathrin-mediated endocytosis
Source: Plant Cell. 2023 Mar 13;35(7):2504–26. doi: 10.1093/plcell/koad069 (PMC10291037; doi:10.1093/plcell/koad069)
Supplement: koad069_Supplementary_Data [file koad069_supplementary_data.zip › TPC2022RA01202DR1_Supplemental Figures and Table.pdf]

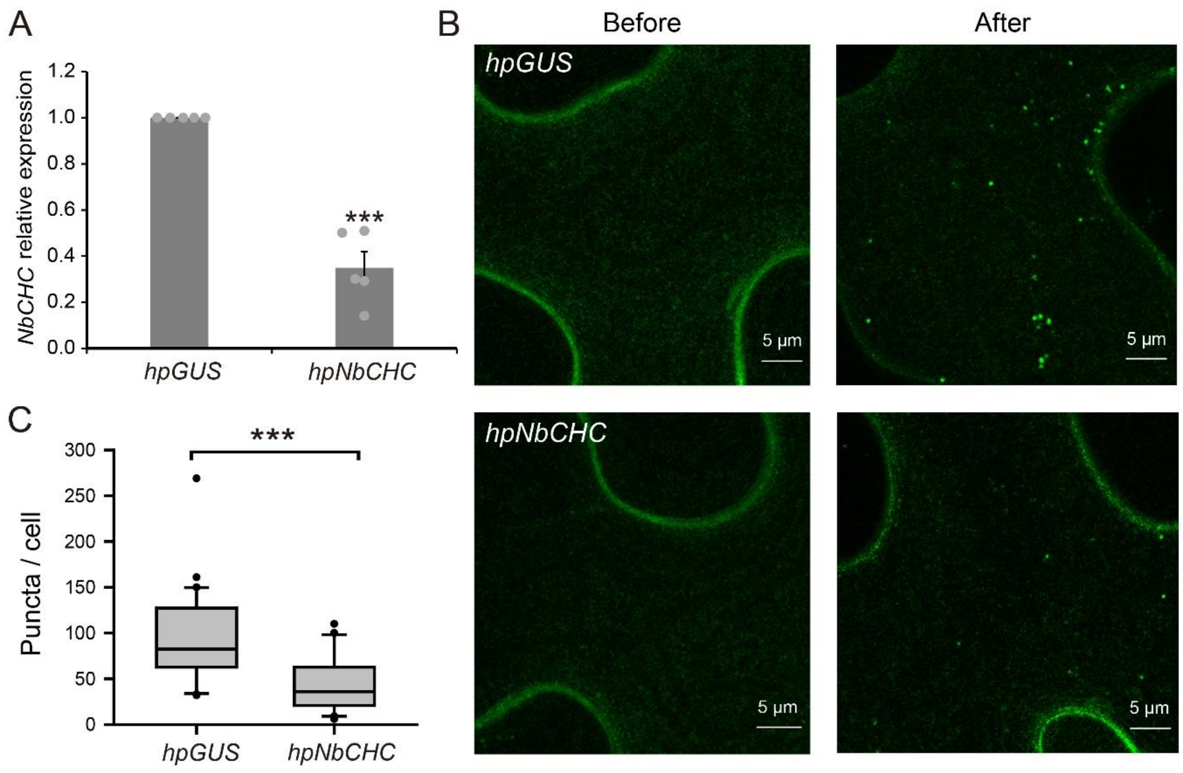

Supplemental Figure S1. Silencing of *NbCHC* reduces flg22-induced endocytosis of FLS2-GFP. (Supports Figure 1.)

(A) *NbCHC* transcript levels relative to control *hpGUS* (which was given a value of 1) as determined by RT-qPCR at 4 d after *N. benthamiana* leaves were infiltrated with a suspension of Agrobacteria carrying *hpNbCHC* (Clathrin heavy chain) or control *hpGUS* constructs. Data shown combines three experimental replicates. Circles represent individual data points. Data are means ± SEM. Significant difference is denoted by asterisks (***$P<0.001$, Student's t-test). **(B)** Representative confocal images of internalized FLS2-GFP endosomes (puncta) in *N. benthamiana* leaf cells expressing *hpNbCHC* or *hpGUS* before and after flg22 peptide treatment. **(C)** Number of internalized FLS2-GFP puncta per cell in *NbCHC*- and control *GUS*-silenced plants after flg22 peptide treatment. One-way ANOVA indicated no significant differences in the behavior of experimental replicates. Combined data are from three experimental replications. A paired two-tailed t-test showed a statistically significant mean difference (*** $P=7.2\times10^{-7}$, n=30 per construct), with a 95% confidence interval of [33, 65].

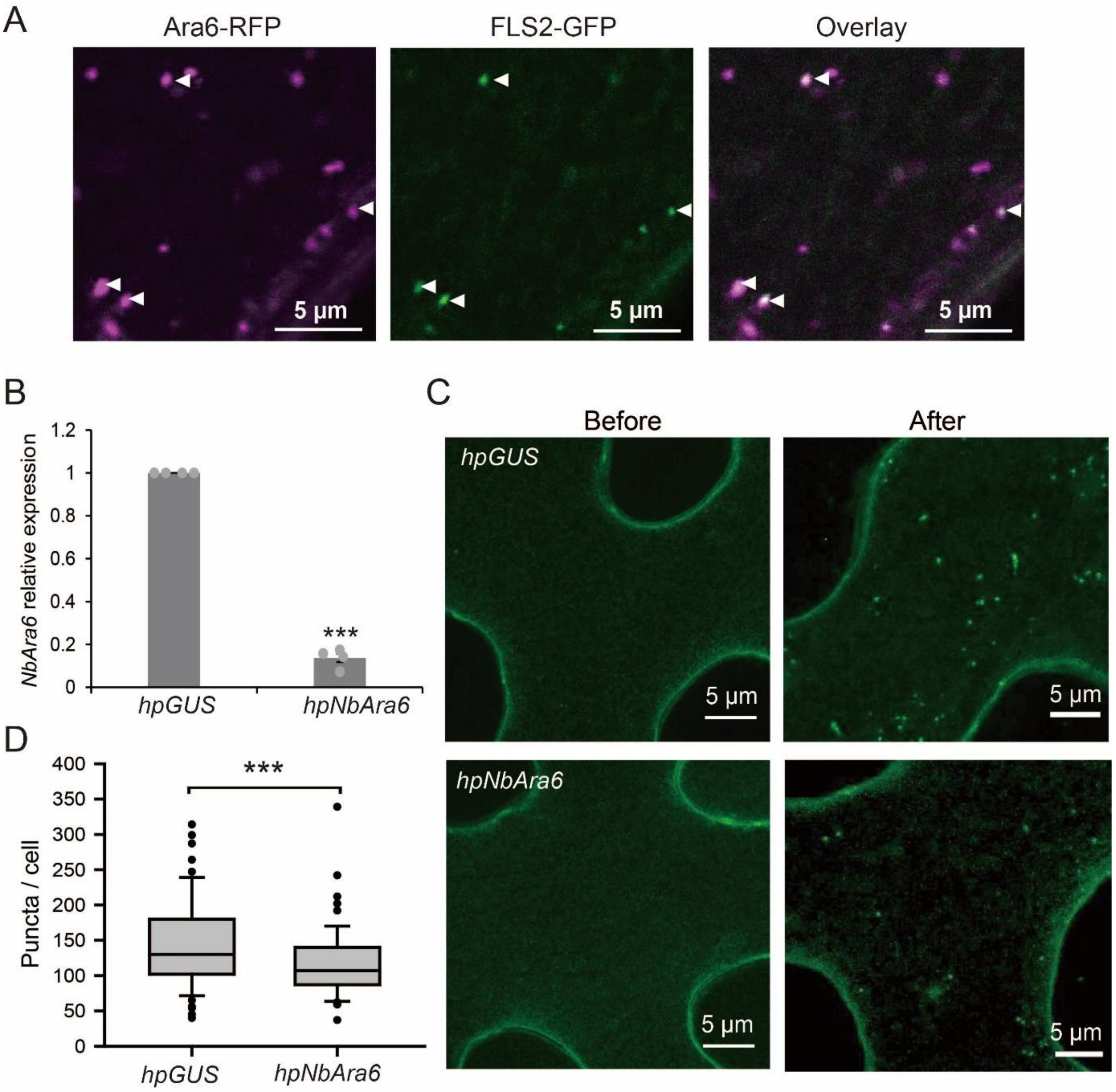

Supplemental Figure S2. Silencing of *NbAra6* reduces flg22 induced endocytosis of FLS2-GFP. (Supports Figure 1.)

(A) Colocalization of internalized FLS2-GFP and early endosome marker Ara6-RFP. Arrowheads indicate example endosomes (puncta) containing both GFP and RFP fluorescence. **(B)** *NbAra6* transcript levels relative to control *hpGUS* as determined by RT-qPCR at 4 d after *N. benthamiana* leaves were infiltrated with Agrobacterium carrying *hpNbAra6* or control *hpGUS* construct. Data shown combines three experimental replicates. Circles represent individual data points. Data are means ± SEM. Significant difference is denoted by asterisks (****P*<0.001, Student's t-test). **(C)** Representative confocal images of internalized FLS2-GFP in *N. benthamiana* leaf cells expressing *hpNbAra6* or *hpGUS*, before or after flg22 treatment. **(D)** Number of internalized FLS2-GFP puncta per cell in *hpNbAra6*- and control *hpGUS*-silenced plants. One-way ANOVA indicated no significant differences in the behavior of experimental replicates. Combined data are from four experimental replications. A paired two-tailed t-test showed a statistically significant mean difference (***P=4.8×10^{-6}, n=57 per construct), with a 95% confidence interval of [18, 41].

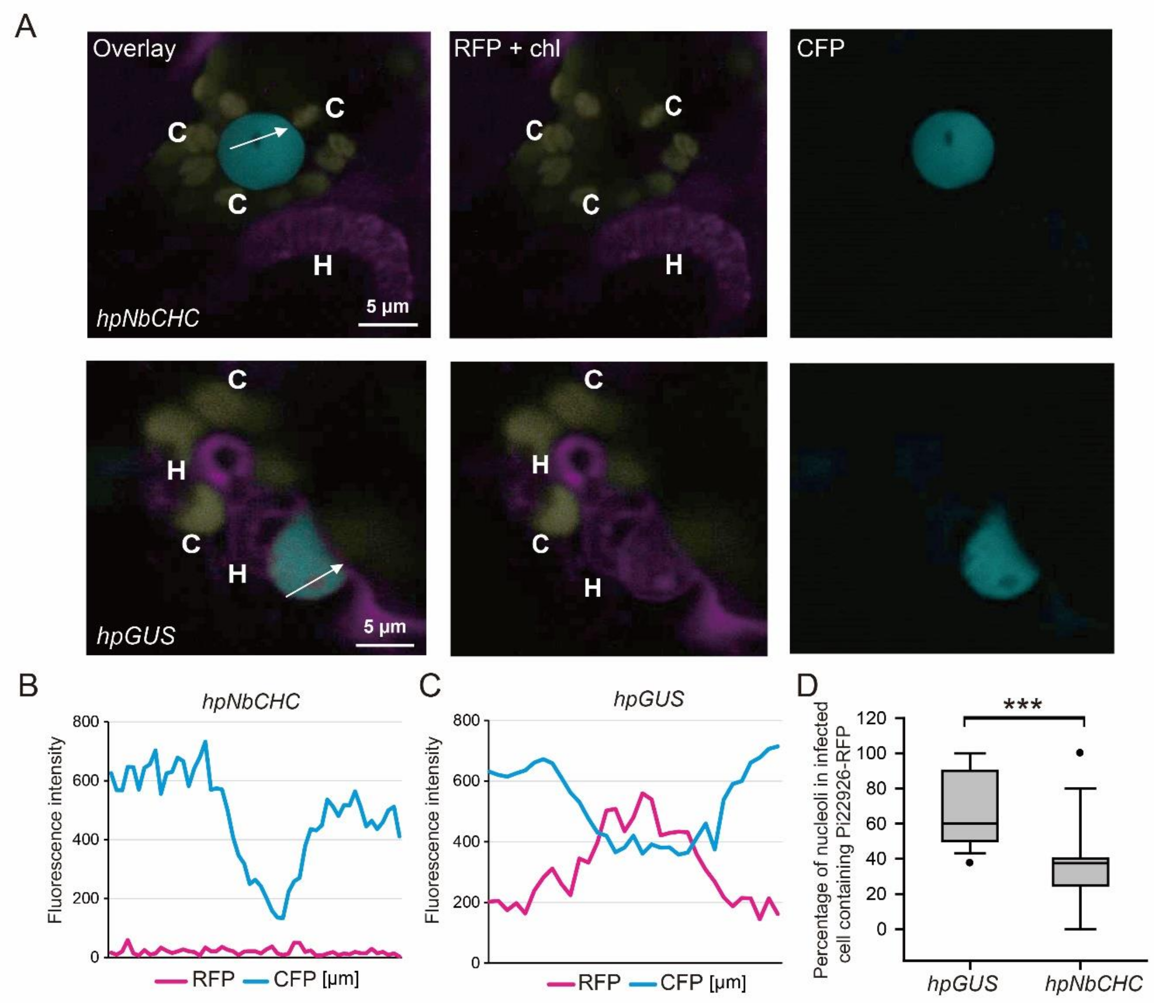

Supplemental Figure S3. Silencing of *NbCHC* reduces RXLR effector Pi22926 accumulation in host nuclei. (Supports Figure 2.)

(A) Representative single optical sections of nuclei near haustoria (H) in infected cells in which the nuclei were labeled by CFP-NbH2B. The nucleolus and nucleoplasm contain red fluorescence, indicating that Pi22926-RFP has translocated from haustoria into the host cell. Chlorophyll autofluorescence “C” is shown in yellow. White arrows show the lines used for the fluorescence intensity profiles indicated in graphs (B) and (C). **(D)** Percentage of host nucleoli containing detectable translocated Pi22926-mRFP of total observed nuclei in infected cells on silenced plants. One-way ANOVA indicated no significant differences in the behavior of experimental replicates. Combined data are from three experimental replications. A paired two-tailed t-test showed a statistically significant mean difference (***$P=3\times10^{-4}$, n=17 per construct), with a 95% confidence interval of [15, 42]. The *x*-axis data in the graphs represents distance (in µm) along each white arrow in the images.

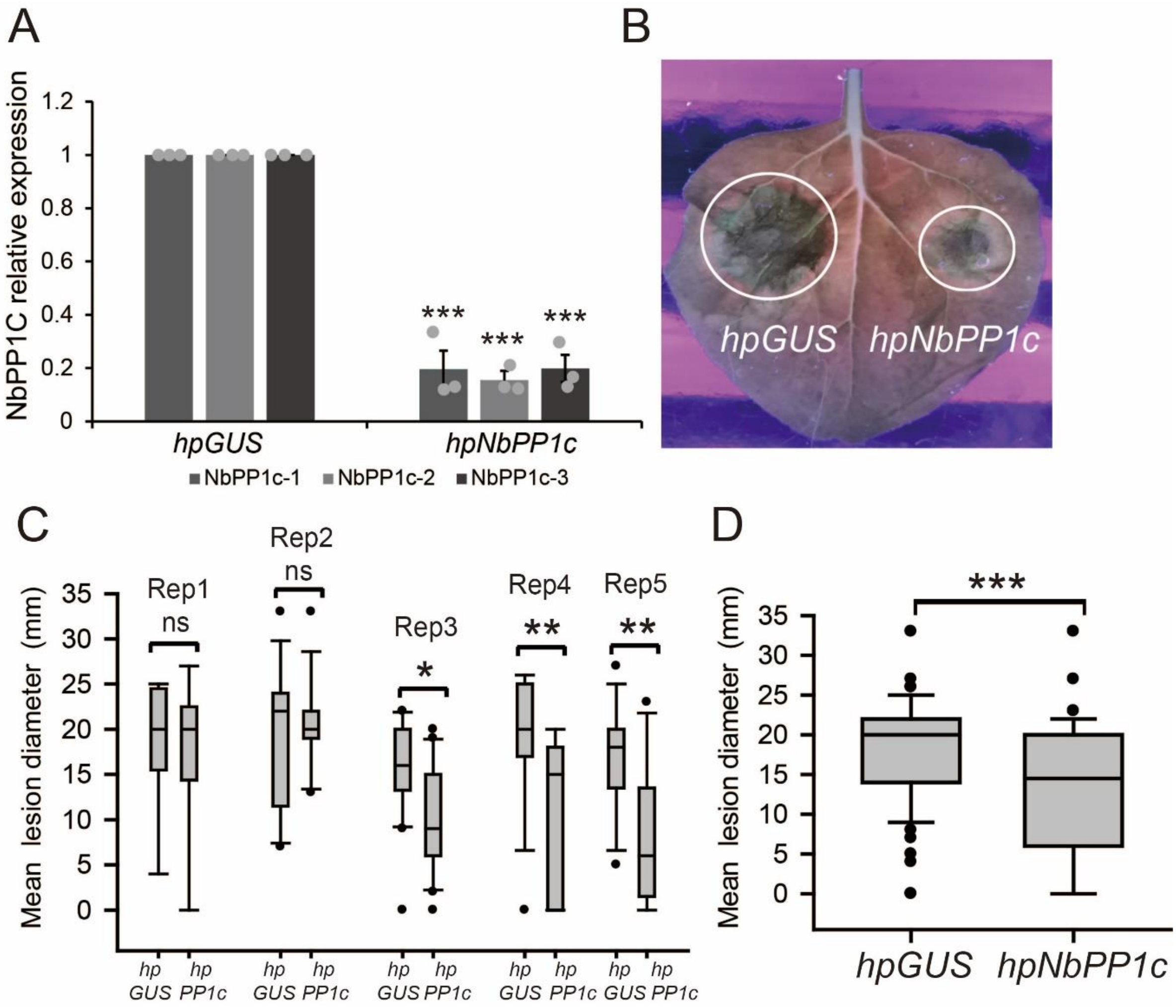

Supplemental Figure S4. Leaf colonization by *P. infestans* is significantly reduced in susceptibility factor *NbPP1C*-silenced plants. (Supports Figure 2.)

(A) *NbPP1C* transcript levels relative to control *hpGUS* as determined by RT-qPCR at 4 d after *N. benthamiana* leaves were infiltrated with Agrobacterium carrying *hpNbPP1c* or control *hpGUS* constructs. Data shown combine three experimental replicates. Circles represent individual data points. Data are means ± SEM. Significant difference is denoted by asterisks (****P*<0.001, Student's t-test). **(B)** and **(C)** Leaf lesion diameter measured at 9 dpi with wild-type *P. infestans* 88069 sporangia suspension combined with infiltration of *hpNbPP1c* and control *hpGUS* in each leaf half. One-way ANOVA indicated there was significant differences between experimental replicates, and so all five independent experimental replicates are shown in (B) and combined data shown in (C). (B) A two-tailed t-test *P*-value was calculated using paired t-test (ns indicates P=0.56, n=13 per construct for Rep1 and *P*=0.56, n=9 per construct for Rep2; asterisks * indicates P=2.5×10^{-2}, n=15 per construct for Rep3; asterisks ** indicates P=5.6×10^{-3}, n=20 per construct for Rep4 and P=5.6×10^{-3}, n=13 per construct for Rep5). With 95% confidence intervals of [–3.4, 6]; [–5.8, 3.3]; [–14, -1.7]; [–9.4, –2.4]; [–14, –3.9], respectively. (C) Combined data are from five experimental replications. A paired two-tailed t-test showed a statistically significant mean difference (*** P=1.8×10^{-5}, n=70 per construct), with a 95% confidence interval of [–7, –2.8]. **(D)** Infected leaf image taken at 9 dpi under UV light.

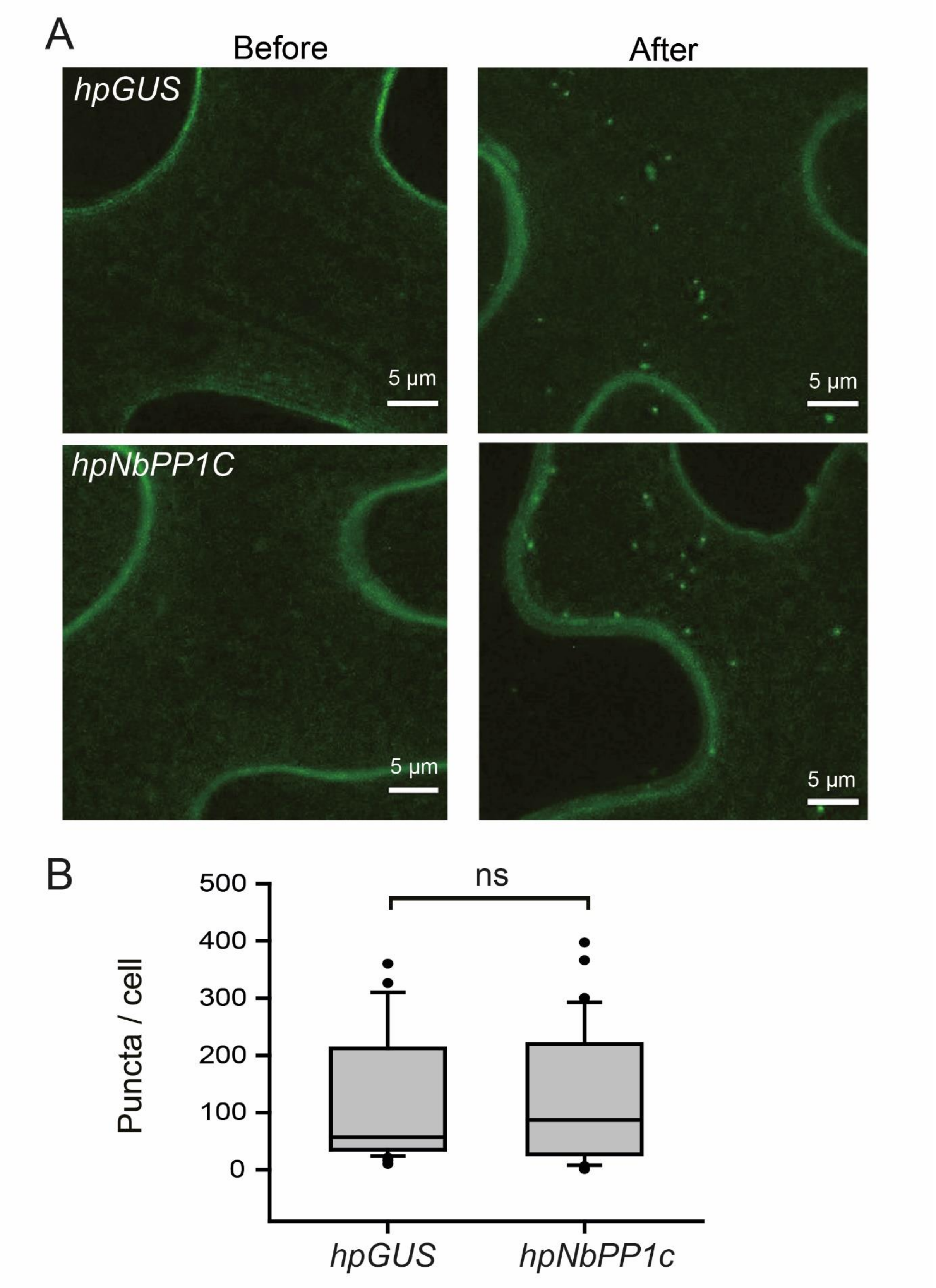

Supplemental Figure5. Silencing of *NbPP1C* does not alter flg22-triggered endocytosis of FLS2-GFP. (Supports Figure 2.)

(A) Boxplot representing the number of internalized FLS2-GFP-positive endosomes (puncta) per cell in *NbPP1C*- and control *GUS*-silenced plants before or after flg22 treatment. One-way ANOVA indicated no significant differences between experimental replicates. Combined data are from three experimental

replications. A paired two-tailed t-test showed a statistically significant mean difference (ns indicates no significant difference, P=0.35, n=36 per construct), with a 95% confidence interval of [–17.8, 6.5]. **(B)** Representative confocal images of internalized FLS2-GFP puncta in *N. benthamiana* leaf cells expressing *hpNbPP1C* or *hpGUS*.

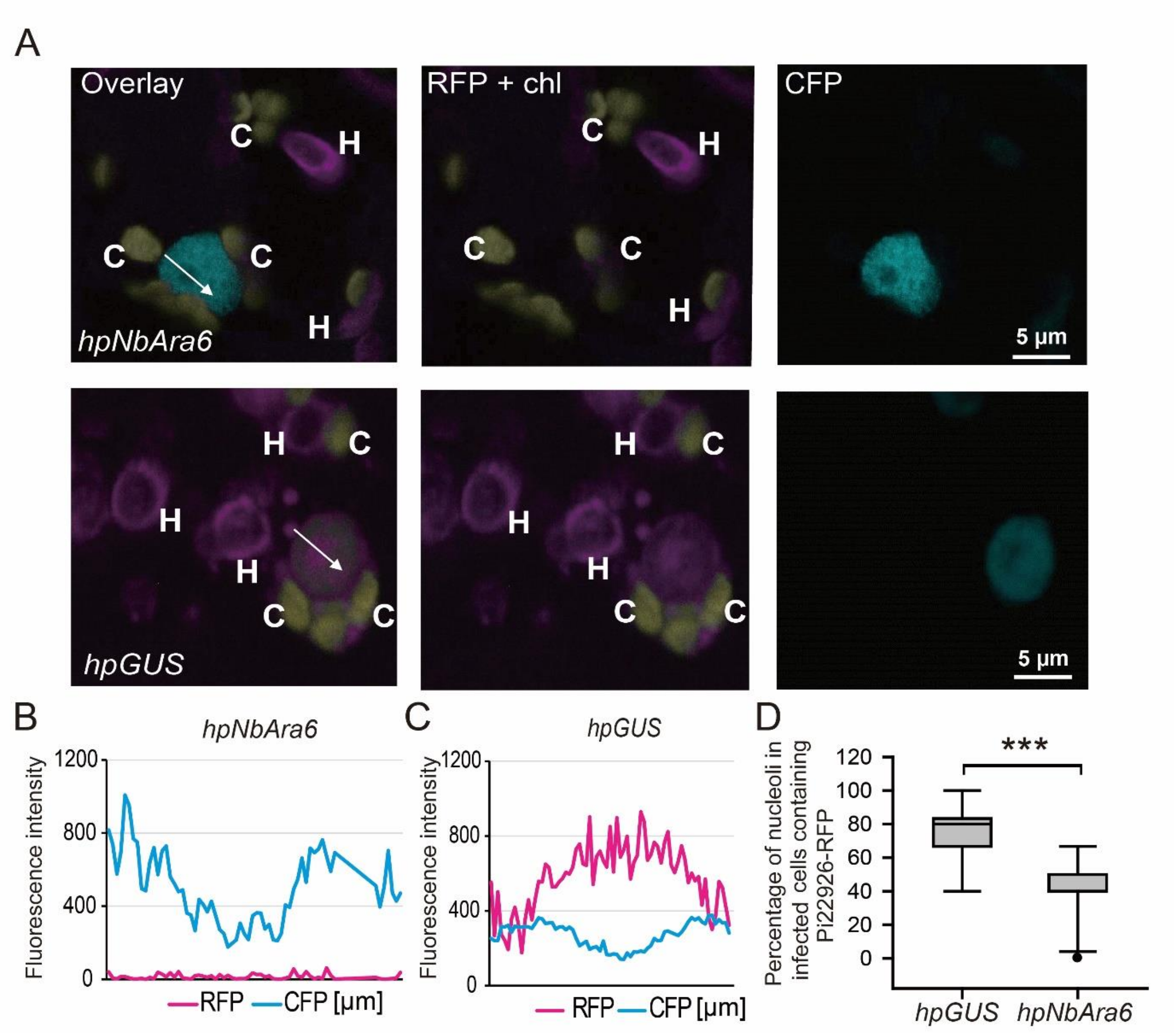

Supplemental Figure S6. Silencing of *NbAra6* reduces RXLR effector Pi22926 accumulation in host nuclei. (Supports Figure 3.)

(A) Representative single optical sections of nuclei near haustoria (H) in infected cells in which the nuclei were labeled by CFP-NbH2B. The nucleolus and nucleoplasm contain red fluorescence, indicating that Pi22926-RFP has translocated from haustoria into the host cell. Chlorophyll autofluorescence “C” is indicated in yellow. The white arrows show the lines used for the fluorescence intensity profiles indicated in graphs for *hpNbAra6* (B) and *hpGUS* (C). **(D)** Percentage of translocated Pi22926-RFP into host nucleoli of infected cells in silenced plants. One-way ANOVA indicated no significant differences between experimental replicates. Combined data are from three experimental replications. A paired two-tailed t-test showed a statistically significant mean difference (***P=5.2×10^{-5}, n=11 per construct), with a 95% confidence interval of [20, 41]. The x-axis data in the graphs represents distance (in µm) along each white arrow in the images.

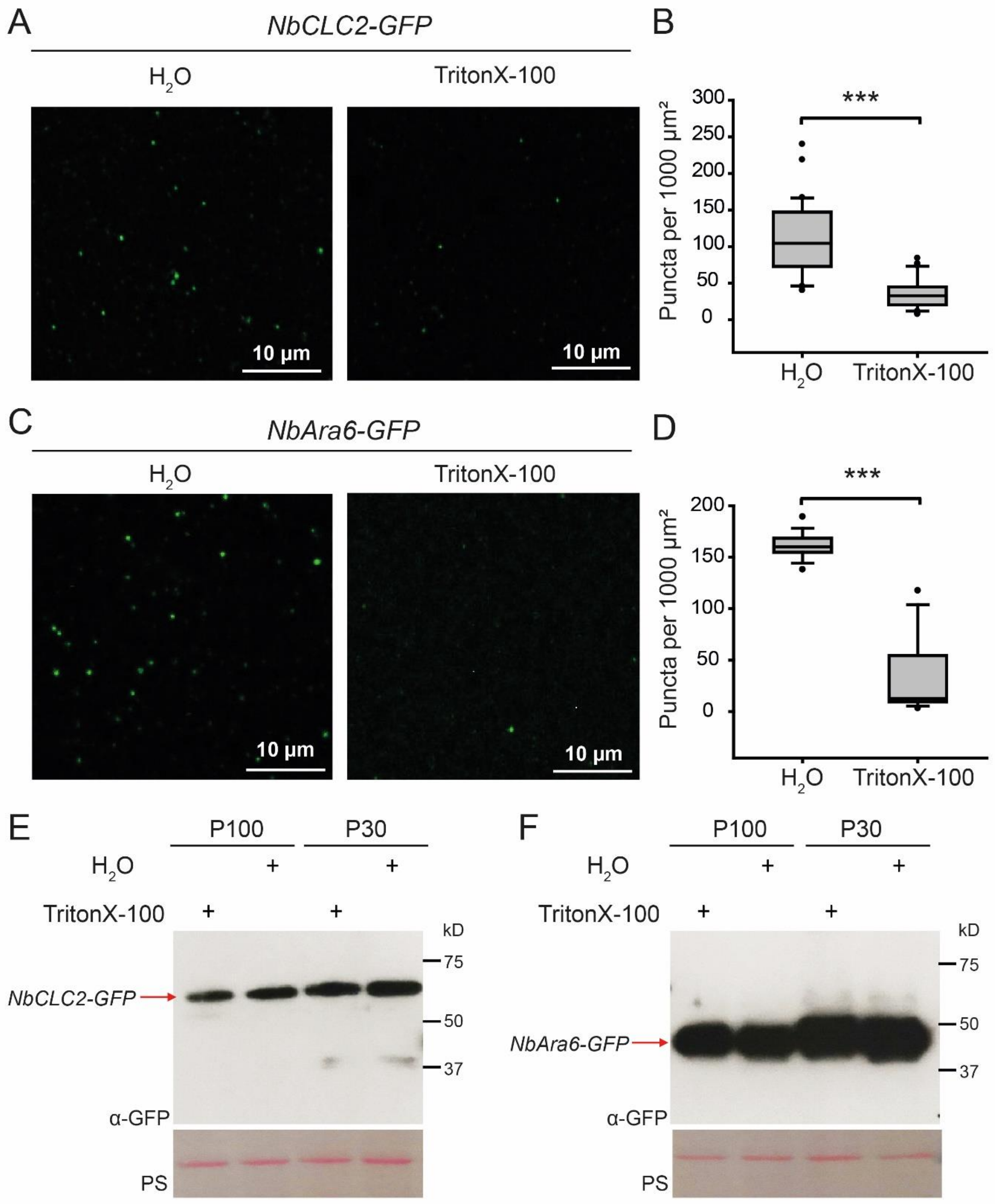

Supplemental Figure S7. P100 enriched puncta are disrupted by Triton X-100 detergent. (Supports Figure 5.)

(A) and **(C)** Maximum intensity projection images of P100 fractions incubated with H_2O or 1% Triton X-100 at room temperature for 1 h. **(B)** and **(D)**, Quantification of NbCLC2-GFP (B, n=2 per treatment) or NbAra6-GFP (D, n=16 per treatment) puncta per 1000 µm^2. One-way ANOVA indicated no significant differences in the behavior of experimental replicates. Combined data are from two experimental replications. Wilcoxon rank sum exact test two showed a statistically significant difference of the means (****P*=6.7×10^{-8}, n=26 per construct for (B) and *P*=1.3×10^{-8}, n=15 for (D)), with a 95% confidence interval of [65, 95] and [123, 152] respectively. NbCLC2-GFP **(E)** and NbAra6-GFP **(F)** were detected by immunoblot using an antibody against GFP (α-GFP) after treatment with Triton X-100 indicating that the proteins were not degraded. Numbers indicate size markers (kD). PS, Ponceau stain.

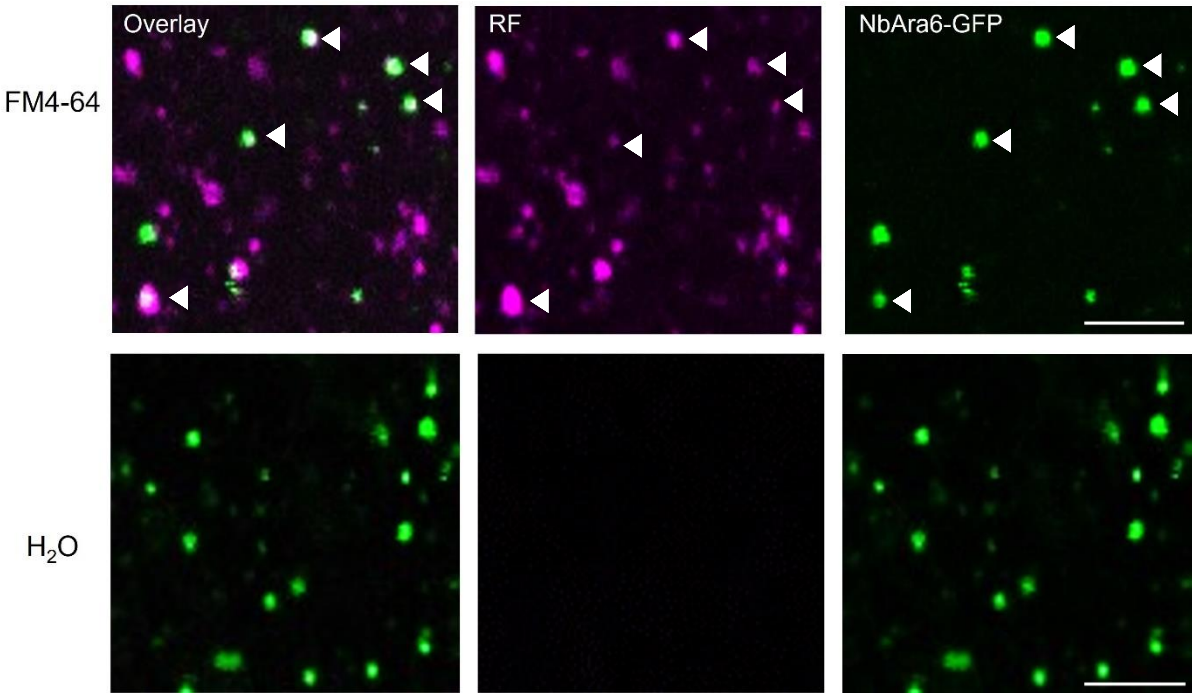

Supplemental Figure S8. Puncta of NbAra6-GFP enriched in P30 fraction stained with FM4-64. (Supports Figure 5.)

Representative maximum intensity projection images of resuspended P30 samples mixed with 8 μM FM4-64 dye, which emits red fluorescence (RF), or with H_2O. Arrowheads indicate example puncta that associated with both fluorophores. Scale bars, 5 μm.

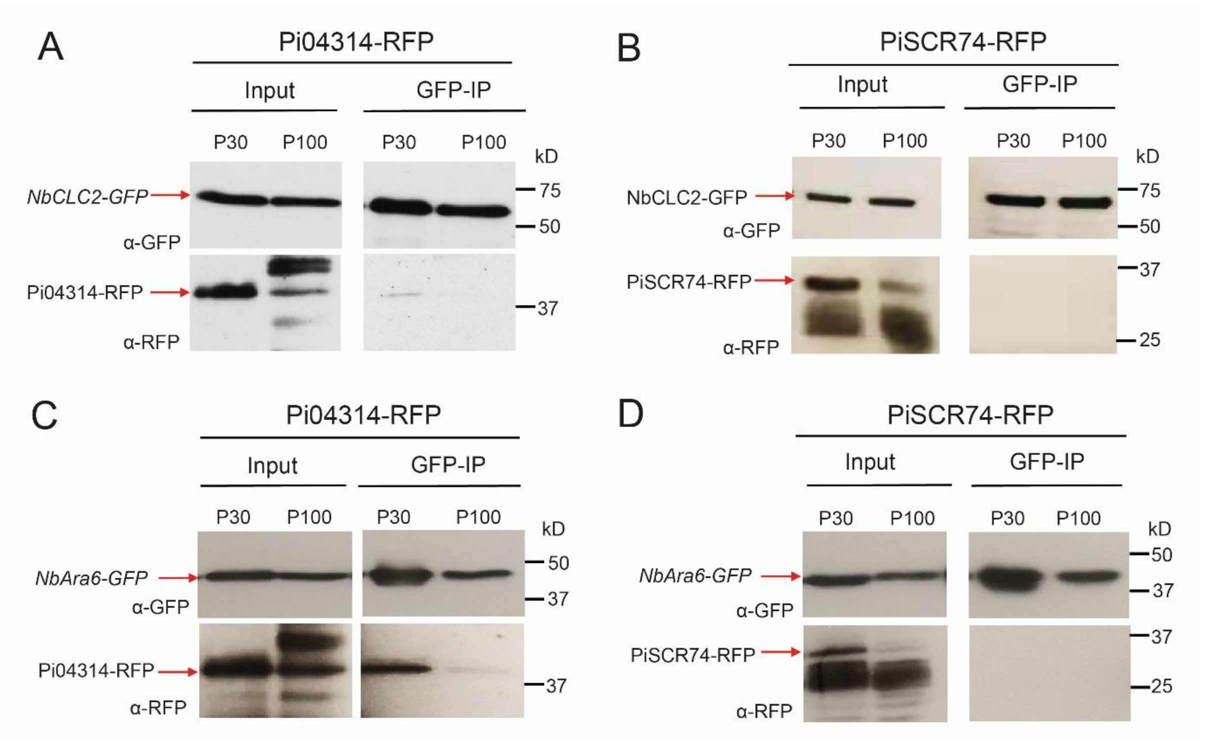

Supplemental Figure S9. The RxLR effector Pi04314 is co-immunoprecipitated with clathrin and Ara6-associated vesicles. (Supports Figure 7.)
(A) and (B), Plant vesicles isolated by ultracentrifugation from leaves infected with *P. infestans* transformants expressing *Pi04314-RFP* (A) or *PiSCR74-RFP* (B) following *NbCLC2-GFP* infiltration. (C) and (D) Plant vesicles isolated by ultracentrifugation from leaves infected with *P. infestans* transformants expressing *Pi04314-RFP* (C) or *PiSCR74-RFP* (D) following *NbAra6-GFP* infiltration. IP samples are purified NbCLC2-GFP- or NbAra6-GFP-labeled vesicles in P30 and P100 samples after incubation with GFP-Trap beads. NbCLC2-GFP and NbAra6-GFP were detected by immunoblot using an antibody against GFP, Pi04314-RFP and PiSCR74-RFP were detected by immunoblot using an antibody against RFP. Numbers indicate size markers (kD).

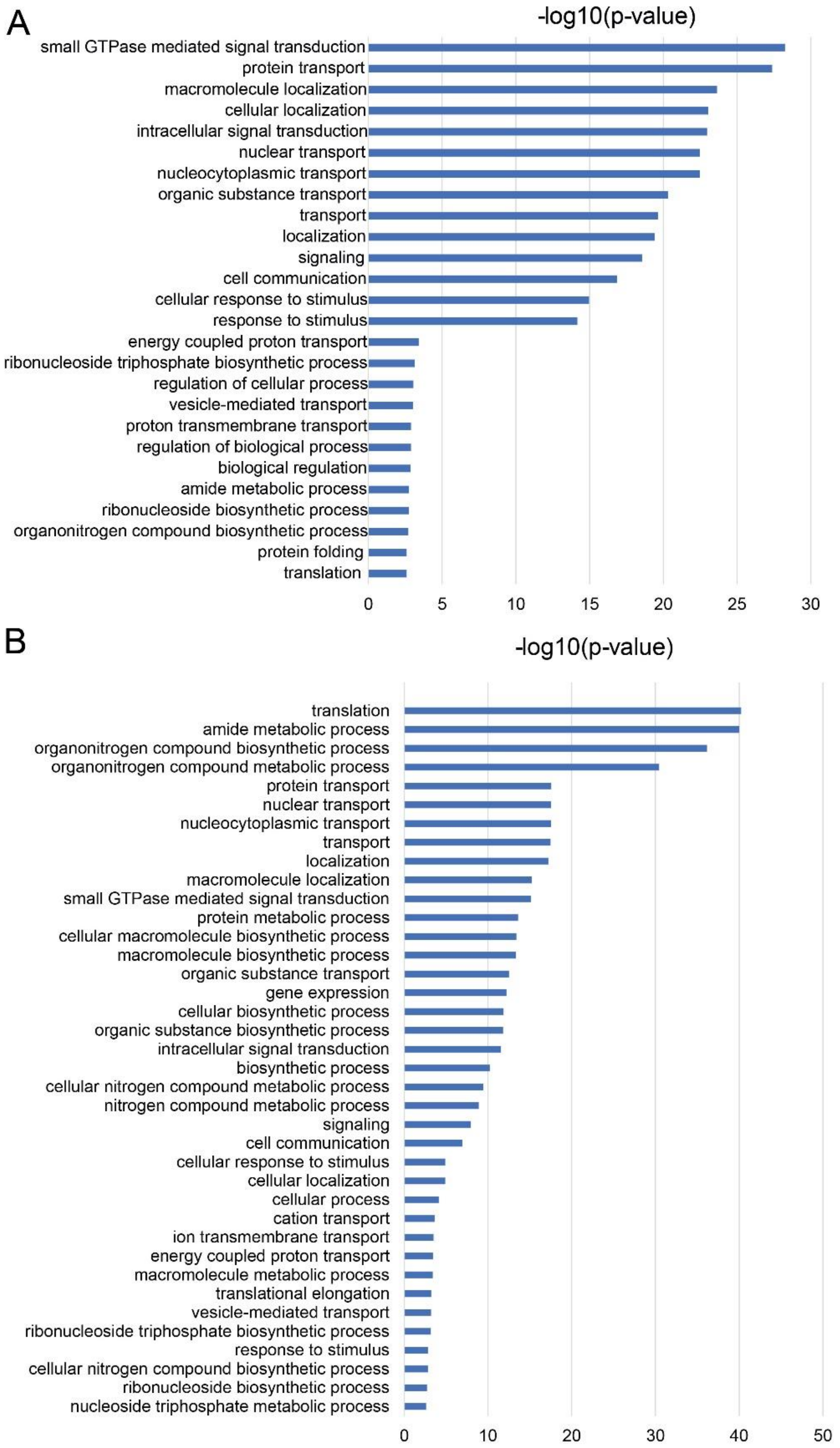

Supplemental Figure S10. Gene ontology (GO) enrichment analysis of proteins significantly enriched in P30-IP and P100-IP following NbAra6-GFP immunopurification. (Supports Figure 8.)
The enriched NbAra6-GFP immunoprecipitated vesicle proteomes from 30,000 × g **(A)** and 100,000 × g **(B)** ultracentrifugation of non-transformed *P. infestans* infected *N. benthmiana* leaves was categorized based on GO terms to biological process through AgriGO v2.0 and Revigo Web site.

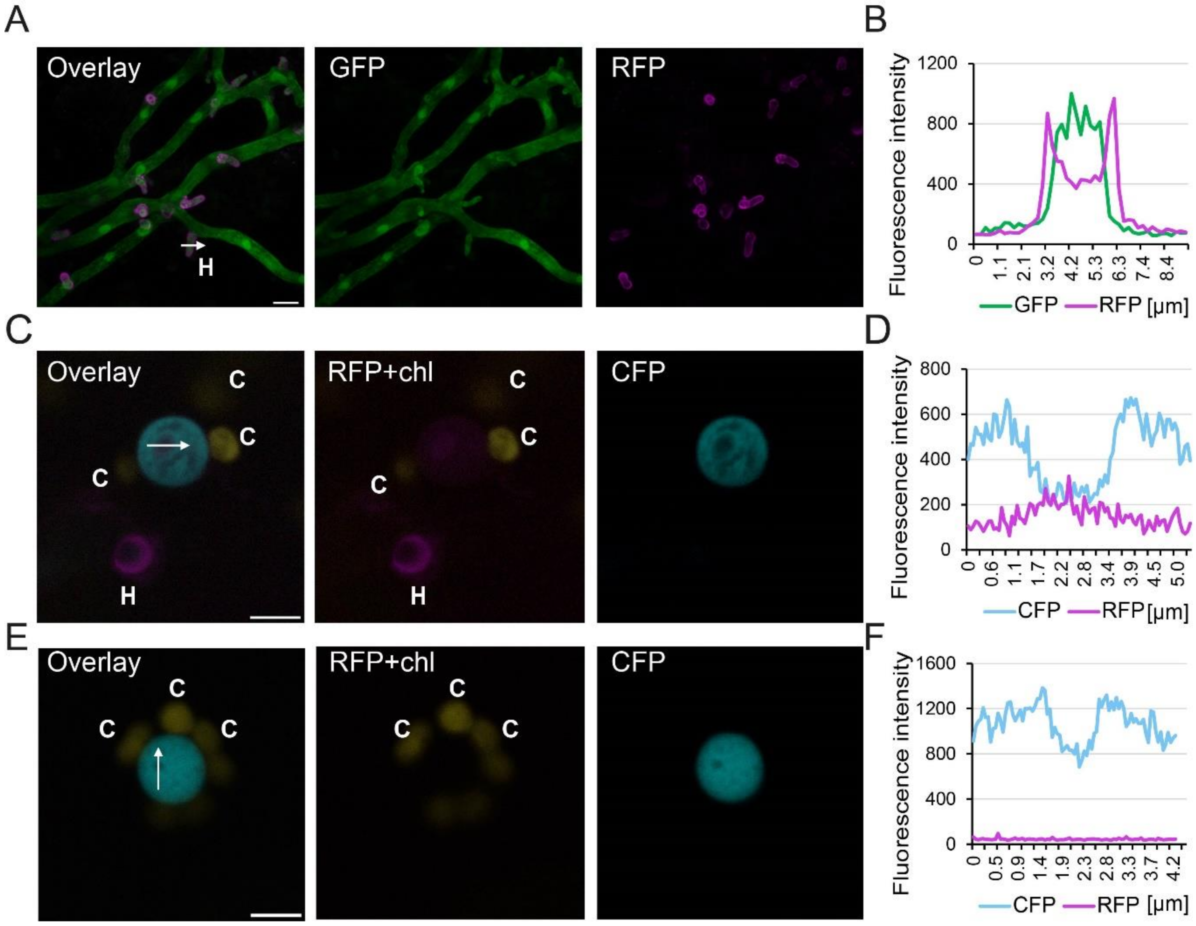

Supplemental Figure S11. Secretion and translocation of RxLR effector AVRblb1 during infection. (Supports Figure 9.)

(A) Representative maximum intensity projection confocal images of *P. infestans* transformant expressing Avrblb1-mRFP and free GFP in the hyphal cytoplasm during infection of *N. benthamiana*. H, haustoria. The white arrow shows the line used for the fluorescence intensity profiles indicated in graph (B). Scale bar, 10 µm. **(C)** Representative single optical sections of nuclei near haustoria (H) in infected cells in which the nuclei were labeled by CFP-NbH2B. The nucleolus and nucleoplasm contain red fluorescence, indicating that Avrblb1-RFP has been translocated from haustoria into the host cell. Chlorophyll autofluorescence “C” is indicated in yellow. Scale bars, 5 µm. The white arrow shows the line used for the fluorescence intensity profile indicated in graph (D). **(E)** Representative single optical sections of nuclei in uninfected cells in which the nuclei were labelled by CFP-NbH2B. Chloroplast autofluorescence “C” is indicated in yellow. Scale bars, 5 µm. The white arrow shows the line used for the fluorescence intensity profiles indicated in graph (F). The x-axis in the graphs represent the distances (in µm) along the length of each white arrow in the images.

Supplemental Table S1. Primers used in this study.

**Primer (5' to 3')**	**Use**
hpGUSF: GGGGACAAGTTTGTACAAAAAAGCAGGCTTCCCAGGCAGTTTTAACGATCAG	Transient silencing
hpGUSR: GGGGACCACTTTGTACAAGAAAGCTGGGTCGATTCACCACTTGCAAAGTCC	
hpNbAra6F: GGGGACAAGTTTGTACAAAAAAGCAGGCTTCTGGGTCAAGGAATTACAAAAACATGGA	
hpNbAra6R: GGGGACCACTTTGTACAAGAAAGCTGGGTCAGACGGGCGCGGTAATCTCTTGGC	
hpNbPP1CF: GGGGACAAGTTTGTACAAAAAAGCAGGCTTCTATAATCAACAGGCTTTTGGAG	
hpNbPP1CR: GGGGACCACTTTGTACAAGAAAGCTGGGTCACTACTTGTTTCCCTGGTTTACC	
NbAra6F: AAAAAGCAGGCTTCACCATGGGTTGCGCATCTTCA	Transient overexpression
NbAra6R: AGAAAGCTGGGTCAGCAGCAGACGGGCGTGGTAA	
NbCLC2F: GGGGACAAGTTTGTACAAAAAAGCAGGCTTCATGTCATCACAGTCAGCACAC	
NbCLC2R: GGGGACCACTTTGTACAAGAAAGCTGGGTCAGGTGCAGCAGTAGCTTGAGG	
qRT-NbAra6-F: ATGGGTTGCGCATCTTCAGTTGC	RT-qPCR primers
qRT-NbAra6-R: TCCTGCAACGCTATTGTTTGAGACA	
qRTNbCHC-F: GCTCAAATTTGTGGGCT AAC AT	
qRTNbCHC-R: GTTGCTGCTCATCACAGGCA	
qRT_Nbef1α-F: AGGCCCTCAGACAAACCCCTCCGT	
qRT_Nbef1α-R: GCTGGATGCTCCCTTGGCTGGGT	
qRTNbPP1c-1F: CAATGTGAGGCTGTGGAAGA	
qRTNbPP1c-1R: ATCGCAAAGCAAACCAGAGT	
qRTNbPP1c-2F: CCTGTGGCAGCACTTATTGA	
qRTNbPP1c-2R: TCGGGTATGGCAGTTGGA	
qRTNbPP1c-3F: CCAGTGGCTGCCTTAATAGATG	
qRTNbPP1c-3R: CCAAAGCAAATCACAGAGCAA	
Avrblb1-NotI-F: GGAAGCGGCCGCACCATGCGTTCGCTCCTGTTG	PCR Amplification
Avrblb1-NotI-R: GGAAGCGGCCGCGGGCTAGGGCCAACGTTT	
